# Signal Amplification for Fluorescent Staining of Single Particles in Liquid Biopsies: Circulating Tumour Cells and Extracellular Vesicles

**DOI:** 10.1002/jev2.70167

**Published:** 2025-10-08

**Authors:** Sara Cavallaro, Sara I. Veiga, Raheel Ahmad, Berent Aldikacti, Mollie Bienstock, Diane Capen, Daniel C. Rabe, Uyen Ho, Dasol Lee, Daniel A. Ruiz‐Torres, Hiroaki Wakimoto, Jorg Dietrich, Brian V. Nahed, Shannon L. Stott

**Affiliations:** ^1^ Krantz‐Family Center for Cancer Research Massachusetts General Hospital Boston Massachusetts USA; ^2^ Department of Medicine Harvard Medical School Boston Massachusetts USA; ^3^ Broad Institute of MIT and Harvard Cambridge Massachusetts USA; ^4^ Shriners Children's Boston Boston Massachusetts USA; ^5^ Program in Membrane Biology/Division of Nephrology Massachusetts General Hospital Boston Massachusetts USA; ^6^ Massachusetts Eye and Ear Massachusetts General Hospital Boston Massachusetts USA; ^7^ Neurosurgery Massachusetts General Hospital Boston Massachusetts USA; ^8^ Brain Tumor Center Massachusetts General Hospital Massachusetts USA

**Keywords:** circulating tumour cells, extracellular vesicles, fluorescence microscopy, liquid biopsies, single particle, signal amplification, tyramide signal amplification

## Abstract

Immunofluorescence (IF) staining represents a convenient and cost‐effective approach to analysing single extracellular vesicles (EVs) and identifying subpopulations with specific roles or biological functions. However, the application of the method is challenged by the weak and unstable signals generated by the low abundant markers carried by the vesicles. In this study, we report the development of an IF strategy based on tyramide signal amplification (TSA) that employs tyramide probes for signal enhancement. The technique is first validated on glioblastoma circulating tumour cells (GBM CTCs) and systematically compared with conventional approaches using fluorescently labelled primary and secondary antibodies. Thereafter, the proposed method is adapted, tested and optimised for the multiplexed fluorescent staining of single EVs isolated from the parental GBM CTCs. The results demonstrate specific staining of single EVs by the developed TSA method, highlighting its advantages of amplified (>6×) signal intensities, more stable signals and broader (∼3×) signal dynamic ranges as compared to the conventional fluorescence methods. The developed protocol also supports multiplexing by incorporating a quenching buffer between the different staining colours. Finally, the protocol demonstrates its applicability to CTCs and EVs derived from plasma samples of GBM patients, with easy adaptation to other cancers or proteins of interest.

## 1 Introduction

Immunofluorescence (IF) staining of single particles poses significant challenges in biological samples due to their inherent heterogeneous composition, often weak signals (Shashkova and Leake 2017), sensitivity to buffer conditions, the necessity for proper sample fixation (Piña et al. 2022), in addition to the possible presence of non‐specific binding (NSB) and elevated background signals (Masters 2012; Shashkova and Leake 2017). These challenges are particularly pronounced in the case of single extracellular vesicles (EVs) (Théry et al. 2018), where the complexities associated with biological samples are compounded by a considerable size heterogeneity (40–2000 nm in diameter) and the very small size of many vesicles (<300–400 nm), most of which fall below the diffraction limit of light (Bordanaba‐Florit et al. 2021; Chiang and Chen 2019; Hilton and White 2021; Welsh et al. 2024). Such a small surface area of the EVs allows for a limited number of targets available for staining, which results in extremely weak signals that are often indistinguishable from the background levels and/or disappear in very short time frames.

Despite these obstacles, the analysis of single EVs is gaining increasing attention and interest due to its pivotal role in resolving the vesicle heterogeneity and, therefore, identifying vesicle subpopulations that might be responsible for specific biological functions (Bordanaba‐Florit et al. 2021; Chiang and Chen 2019; Hilton and White 2021; Spitzberg et al. 2023; Ter‐Ovanesyan et al. 2017). This type of information cannot be obtained by using bulk analytical methods, as such approaches can only analyse average properties (Bordanaba‐Florit et al. 2021; Erdbrügger and Lannigan 2016; Ferguson et al. 2022; Ter‐Ovanesyan et al. 2017). Given the significance of single vesicle analysis but considering the challenges posed by the limited cargos of these biological particles, recent efforts have focused on the meticulous development and/or optimization of techniques to amplify the signals and/or increase the sensitivity/limit of detections (Guo et al. 2021; Yang et al. 2021). Among all the methods available to characterise EVs at a single particle level, IF represents an efficient, convenient and versatile approach to visualise the cargos on the vesicles, semi‐quantify them and compare them between various samples (He et al. 2023; Higginbotham et al. 2011; Spitzberg et al. 2023; Wardhani et al. 2024; Welsh et al. 2024). The method also offers the advantage of limited costs and the possibilities for automation and multiplexing (Francisco‐Cruz et al. 2020; He et al. 2023).

To overcome the aforementioned challenges in single EV analysis and considering the advantages as well as the challenges of IF methods, in this work, we focus on the development of a signal amplification strategy based on IF staining. Specifically, the developed protocol is based on tyramide signal amplification (TSA), and herein named TSA, as it utilises small reporter molecules called tyramide probes to obtain signal enhancement. Briefly, the TSA method relies on the horseradish peroxidase (HRP) that is conjugated to a secondary antibody to enzymatically convert an unreactive tyramide fluorescent probe to a reactive form. Once activated, the fluorescent tyramide covalently binds the tyrosine residues that are on and surrounding the protein epitope targeted by the primary antibody. Since a single HRP‐conjugated secondary antibody can activate multiple tyramide probes, the fluorescent signal is significantly amplified compared to conventional staining methods using directly labelled fluorescent antibodies. This approach improves the detection of low‐abundance markers, providing increased signal‐to‐noise (SNR) measurements (Faget and Hnasko 2015; Shojaeian et al. 2018; Stack et al. 2014). Moreover, in comparison to conventional staining methods employing fluorescently labelled primary or secondary antibodies, the TSA technique exhibits minimal non‐specific staining, broader signal dynamic ranges and enhanced signal stabilities over time (Gong et al. 2012; Wang et al. 2023), while maintaining comparable costs to the other techniques (Stack et al. 2014). All these features assume critical importance in the context of staining single EVs, given the very low abundance of many cancer‐specific markers carried by the vesicles and their often very poor signal stabilities (Hilton and White 2021; Ko et al. 2020).

Although TSA approaches have been used in several cases for the staining of cells (Chen et al. 2020; Roy et al. 2019), their specific utilization in circulating tumour cells (CTCs) has been limited (Zhou et al. 2019), and even less so in the context of analysis of EVs. Glioblastoma, the most common and aggressive form of malignant brain tumour with a remarkably poor prognosis (5‐year survival rate <5%) (Lah et al. 2020; Stupp et al. 2009), is a primary focus in our laboratory, which has a longstanding history in the analysis of CTCs and EVs from GBM patients (Reátegui et al. 2018; Zachariah et al. 2018). Existing literature on TSA analysis has predominantly focused on whole tissue staining (Alban et al. 2018; Bloch et al. 2013; Liao et al. 2021; Parra et al. 2020; Roy et al. 2019; Stack et al. 2014; Toda et al. 1999), using fixed, paraffin‐embedded sections mounted on glass slides, providing structural integrity as well as ample protein fixation. Further, works focusing on CTCs have been mostly centred around other cancer types, such as prostate, colorectal and adenocarcinoma (Deelstra 2017; Ntouroupi et al. 2008). Tubbs et al. developed a TSA‐fluorescence in situ hybridization (TSA‐FISH) method for staining a single marker on GBM cancer cells (Tubbs et al. 2007), while a few other studies have utilised similar methods, based on commercially available kits, for tissue staining rather than for the analysis of single CTCs from GBM patients (Alban et al. 2018; Cloughesy et al. 2019; Hernandez et al. 2021). To the best of our knowledge, a systematic and comprehensive exploration of TSA technique in comparison to conventional staining methods for single CTC analysis, particularly in the realm of GBM CTCs, is notably lacking in the current literature. In a previous work led by our collaborator, Dr. Brian Nahed (Sullivan et al. 2014), we have shown that a specific antibody cocktail can differentiate GBM CTCs from white blood cells (WBC) using direct fluorescent staining. Building upon this work, in this study, we first validate the developed TSA protocol on CTCs. Specifically, we demonstrate the possibility of staining and discriminating GBM CTCs from WBC using the identical GBM CTC antibody cocktail, now integrated into a tyramide‐based signal enhancement mode. Furthermore, we provide a systematic comparison with conventional staining methods, including direct and secondary antibody staining, shedding light on the efficacy and potential advantages of the TSA approach in the intricate analysis of single CTCs.

Thereafter, we show the validation and application of the developed TSA method for the staining of single EVs isolated from the aforementioned parent GBM CTCs. The few existing reports using a TSA approach have primarily applied it to stain EVs in bulk (Chen et al. 2021; Chen et al. 2019; Jo et al. 2023), with only very limited instances of applying this method to label single vesicles. Up to date, there are only three studies, conducted by Nguyen et al. (2022), Chen et al. (2022) and Reynolds et al. (2023), that have reported the use of a tyramide amplification strategy for the fluorescence labelling of single EVs. However, these investigations do not include signal multiplexing and have limited validation of the method. In this study, we focus on the development of a TSA protocol that is specifically tailored for the staining of single vesicles and can be multiplexed. The proposed method is cost‐effective as it can be replicated without the need to buy expensive commercial kits, such as the Opal kits. Moreover, we aim to provide a systematic comparison between the developed single EV TSA method and the conventional methods using fluorescently labelled primary and secondary antibodies. This comparative analysis seeks to elucidate the respective advantages and disadvantages of each technique, empowering researchers to make informed decisions based on their specific application requirements and unique needs when staining individual EVs.

## 2 Materials and Methods

### 2.1 Materials and Reagents

Phosphate‐buffered saline (PBS, cat. # 10010–023) pH 7.4 solution, Alexa Fluor 488 Tyramide Reagent (cat. # B40953, TSA‐AF488), Alexa Fluor 594 Tyramide Reagent (cat. # B40957, TSA‐AF594), Anti‐A2B5 antibody (cat. # MA1‐90445), Anti‐CD11c antibody (cat. # 14‐0116‐82), Goat Anti‐Mouse IgG1‐AF594 antibody (cat. # A‐21125), Goat Anti‐Mouse IgG2b‐AF488 antibody (cat. # A‐21141), Anti‐EGFR‐AF488 antibody (cat. # MA5‐44134), DAPI (cat. # D1306) and ProLong Glass Antifade Mountant (cat. # P36980) were purchased from ThermoFisher Scientific. UltraPure 1M Tris‐HCI pH 8.0 solution (cat. # 15568‐025) was purchased from Invitrogen. NaCl powder (cat. # S3014), Triton X‐100 solution (cat. # T8787), Anti‐Sox2 antibody (clone 10H9.1, cat. # MAB4423) and 3% Hydrogen Peroxide solution (cat. # 88597) were purchased from Millipore Sigma. TSA blocking agent (cat. # FP1020), Antibody Diluent/Block (100 mL, cat. # ARD1001EA) and 1× Plus Automation Amplification Diluent (50 mL, cat. # SKU FP1609) were obtained from Perkin Elmer. Goat α‐Mouse IgG1 Fab Fragment (cat. # 115‐007‐185), Goat α‐Rat IgG Fab Fragment (cat. # 112‐007‐008), Anti‐Mouse IgG2b HRP antibody (cat. # 115‐035‐207), Anti‐Mouse IgM HRP antibody (cat. # 115‐035‐020), Anti‐Rat IgG2b HRP antibody (cat. # 112‐035‐143) and Anti‐Mouse IgG1 HRP antibody (cat. # 115‐035‐205) were purchased from Jackson Laboratory. Anti‐beta‐III Tubulin antibody (clone CL5814, cat. # NBP2‐61‐431), Anti‐c‐MET antibody (clone OTI1E6, cat. # NBP2‐45822), Anti‐CD63 antibody (cat. # NB100‐77913), Anti‐CD63‐AF594 antibody (cat. # NB100‐77913AF594) and Anti‐cMET antibody (cat. # FAB3582G) were purchased from Novus Biological. Anti‐EGFR antibody (cat. # ab30) was purchased from Abcam. Anti‐CD9 antibody (cat. # 312102) and Anti‐CD81 antibody (cat. # 349502) were purchased from BioLegend. Anti‐CD45 antibody (cat. # sc70699) was purchased from Santa‐Cruz Bio. If not stated otherwise, all the other chemicals were purchased from Millipore Sigma.

### 2.2 Cell Culture of Glioblastoma Primary Cells

Two suspension glioblastoma cell lines were used for this work. The first is MGG72, a GBM stem‐like cell line derived from a patient primary tumour that was established at Massachusetts General Hospital by Wakimoto et al. (Saha et al. 2017; Wong et al. 2015). For the sake of simplicity, in this study, we refer to this cell line as GBM1. In addition to MGG72, we also used a cell line that was established from CTCs isolated from the blood of a GBM patient, here referred to as GBM2 (Rabe et al., in preparation). For the GBM1 cells, Neurobasal medium (cat. #21103, Gibco/Invitrogen) supplemented with L‐glutamine, B27 (cat. # 17504‐044, ThermoFisher Scientific) and N2 (cat. # 17502048, ThermoFisher Scientific) supplements, epidermal growth factor (EGF, cat. # 100–26, FUJIFILM Irvine Scientific), fibroblast growth factor (FGF, cat. # 100–146, FUJIFILM Irvine Scientific), Penicillin/Streptomycin (cat. # 10378016, ThermoFisher Scientific) and Heparin (cat. # 07980, STEMCELL Technologies) was used for culture. GBM2 CTCs were cultured in hypoxic conditions, using RPMI‐1640 1× with L‐glutamine medium (cat. # 10‐040‐CV, ThermoFisher Scientific) supplemented with N21‐Max (cat. # AR008, R&D Systems), EGF, FGF, antibiotic‐antimycotic and Heparin. For the experiment requiring fluorescently labelled EVs, the parent GBM2 CTCs were lentivirally transduced with a palmitoylated‐tdTomato fluorescent reporter, as described by (Rabe et al. 2023).

### 2.3 GBM Primary Cell Plating

The GBM cells were obtained by spinning the cell culture media at 300 × *g* for 5 min at room temperature (RT). The cell pellet from this first spin was resuspended in 1× PBS and centrifuged again at 300 × *g* for 5 min at RT. After this second spin, the cell pellet was resuspended again in 1× PBS, and the cells were counted under an automated cell counter (Auto T4, Nexcelom Biosciences). Two resuspensions of the cell pellet in PBS were used to decrease the presence and fluorescence of the Phenol Red component included in the cell culture media, which could interfere with the staining. Following the two rounds of spins, a specific number of GBM cells (20k or 50k, depending on the experiment) was fixed using paraformaldehyde (PFA, cat. # 28906, ThermoFisher Scientific; 1.1% PFA solution in 1× PBS for 10 min) and plated onto TruBond380 adhesive glass slides (cat. # 50‐340‐33, Fisher Scientific) using a cytospin (2000 rpm for 5 min, low acceleration). Following cytospin, the plated GBM cells on the slides were further fixed in a 2% PFA solution (for 10 min), washed with 1× PBS and deionised (DI) water, dried in air and stored at −80°C until use. On the day of the staining experiments, the GBM cell slides were thawed at RT and stained with a fluorescence staining protocol.

### 2.4 Extracellular Vesicle Isolation

The GBM EVs used in this study were obtained from the cell‐conditioned media of the two‐parent primary GBM cell lines, GBM1 and GBM2. Both EV samples were purified and isolated using a combination of centrifugations, concentration and size exclusion chromatography (SEC). Specifically, for each experiment the GBM1 EVs were isolated from two T75 flasks containing approximately 2.7 × 10^6^ total GBM1 cells and 30 mL total cell conditioned media, while the GBM2 EVs were isolated from two T75 flasks containing approximately 4.5 × 10^6^ total GBM2 cells and 30 mL total cell conditioned media. For each cell line, the 30 mL of cell‐conditioned media was first spun at 300 × *g* for 5 min at RT to remove cells, then spun at 2000 × *g* for 10 min at RT to remove cell debris and aggregates, and finally concentrated to 500 µL using a 4000 × *g* spin for 20 min at RT in Amicon 15 mL centrifugal filters (MWCO 10 kDa, cat. # UFC9010, Millipore Sigma). Following centrifugation and concentration, the concentrated EV samples were isolated using Izon qEVoriginal 70 nm Gen2 columns (cat. # ICO‐70, Izon Science Ltd), following the protocol recommended by the manufacturer. Briefly, after initial washings of the columns with water and/or 1× PBS, 500 µL of concentrated EV sample was pipetted into each column, and 1.6 mL of purified EV sample in PBS was collected from each outlet. Following sample collection, the columns were washed with a sequence of NaOH, water and 20% EtOH. On the same day of isolation, the EVs were immobilised on top of the TruBond380 (TB380) glass slides by using overnight adsorption. The following day, fluorescence staining of the immobilised EVs was performed.

### 2.5 Nanoparticle Tracking Analysis

The size and concentration of the isolated EV samples were characterised using nanoparticle tracking analysis (NTA). Specifically, a Nanosight 3000 (Malvern Instruments) and a ViewSizer 3000 (HORIBA Scientific) were used for the measurements. For the Nanosight 3000 experiments, five videos of 30 s each (camera level: 12, detection threshold: 5) were recorded and processed for all samples. For the ViewSizer 3000 experiments, 25 videos of 30 s each (exposure: 15 ms; frames per second: 30) were recorded and processed for each sample, using the following laser powers: 210 mW for the 455 nm laser, 12 mW for the 520 nm laser and 8 mW for the 635 nm laser.

### 2.6 Western Blotting

GBM2 parent CTC cell pellets and isolated EVs were lysed in 1× RIPA lysis buffer (Millipore Sigma, cat. # 20–188) supplemented with 1× protease inhibitors (cOmplete Protease Inhibitor Cocktail, cat. # 11697498001). Lysed samples were mixed with Laemmle sample buffer (1% final concentration) containing 2‐mercaptoethanol (350 mM) to reduce disulfide bonds in proteins and heated to 95°C for 5 min. For each sample, ∼3 µg of protein was loaded onto a precast 4%–20% polyacrylamide gel (Mini‐PROTEAN TGX, Bio‐Rad, cat. # 4561094) and then transferred to methanol‐activated polyvinylidene difluoride (PVDF) membranes. Further, the membranes blocked for 1 h with 5% skim milk dissolved in 1× Tris‐buffered saline containing 0.1% Tween 20 (TBST). The membranes were incubated overnight with primary antibodies (1:500 dilutions in 5% BSA with 0.02% sodium azide prepared in 1× TBST): HSP70 (cat. # 4872, Cell Signalling), CD63 (cat. # NB100‐77913, Novus Biologicals) and CD9 (cat. # 312102, BioLegend). The membranes were then washed three times with TBST buffer and incubated for 1 h with HRP‐bound secondary antibodies (1:5000 dilutions): Anti‐rabbit IgG (cat. # 7074), Anti‐mouse IgG (cat. # 7076), depending on the source of the primary antibody. Membranes were washed three times with TBST buffer before incubation with enhanced chemiluminescent substrates (Pierce ECL Western Blotting Substrate or SuperSignal Western Blot Enhancer, ThermoFisher Scientific, cat. # 34580 and 46640) depending on the protein transferred onto PVDF membranes. Images were acquired in the darkroom with different exposures times on autoradiography films (Hyblot CL Autoradiography Film, Thomas Scientific, cat. # 11441J51).

### 2.7 Enzyme‐Linked Immunosorbent Assay (ELISA)

Protein levels on the EVs were accessed for EGFR (cat. # ELH‐EGFR, RayBiotech) and CD9 (cat. # OKCD00751, Aviva Systems Biology) using the ELISA commercial kits from the corresponding companies. The assays were performed according to the manufacturer's protocols.

### 2.8 Transmission Electron Microscopy

Aliquots (10 µL) of freshly isolated EV suspensions (GBM2 EVs) or suspensions that had previously been stored at −80°C (GBM Pt3 and Pt4), were applied undiluted onto 200 mesh Formvar/carbon coated nickel grids (Electron Microscopy Sciences, Hatfield, PA) and allowed to adsorb for 15 min. The grids were then blotted (to remove excess suspension), briefly rinsed once with filtered distilled deionised water, and contrast‐stained for 10 min in a tylose/uranyl acetate solution on ice. Thereafter, the grids were blotted again and allowed to air dry prior to analysis. Examination of the preparations was performed using a JEOL JEM 1011 transmission electron microscope at 80 kV. Images were collected using an AMT digital imaging system with proprietary image capture software (Advanced Microscopy Techniques, Danvers, Massachusetts).

### 2.9 dSTORM Imaging of EVs

The tetraspanin (CD9, CD63 and CD81) composition of the EVs was further investigated using the dSTORM Nanoimager system (ONI). For these measurements, the EV Protein Profiler kit (cat. # EV‐MAN‐1.0, ONI) was used. The EVs were immobilised into the inner surface of a functionalised ONI microfluidic chip using a cocktail of CD9, CD63 and CD81 capture antibodies, and following the immobilization protocol recommended by the manufacturer. After functionalization of the microfluidic devices, capture and fixation of the EVs, the tetraspanins on the surface of the vesicles were targeted using a combination of CD9‐AlexaFluor488 (CD9‐AF488), CD63‐AlexaFluor555 (CD63‐AF555) and CD81‐AlexaFluor630 (CD81‐AF630) fluorescent antibodies, following the staining protocol recommended by the manufacturer. The stained vesicles were then imaged under the ONI microscope, and their tetraspanin composition was analysed on single particles using CODI software, following the image analysis guidelines recommended by the manufacturer.

### 2.10 EV Immobilization on Glass Slides

For the staining experiments performed in this study, the EVs were mostly immobilised onto TruBond380 adhesive glass slides using overnight adsorption. In this method, the EVs diluted in PBS isolated from the Izon columns were incubated overnight on top of a TB380 glass slide mounted with a MilliCell insert chamber (cat. # PEZGS0416, Millipore Sigma). The following day, the unbound vesicles were washed off using 1× PBS and the immobilised EVs were stained using the specific fluorescent staining protocol. At the end of the protocol and before imaging, the stained vesicles onto the slide were covered using a glass coverslip (170 µm thickness, cat. # 2980‐245, Corning) and PBS as a ‘mounting media’. This strategy avoided drying of the vesicles, which could cause deterioration of the signals and detachment of the vesicles from the surface due to high surface tension forces. For the initial validation experiments, some of the EV samples were immobilised onto the glass slides using two additional strategies: adsorption onto a Poly‐L‐Lysine (PLL) glass slide (cat. # P0425, Millipore Sigma) and cytospin immobilization onto either PLL or TB380 glass slides. For the adsorption onto the PLL slides, the same method as the TB380 adsorption was performed. For the immobilization using cytospin, the vesicles were immobilised onto the respective slide using fixation with PFA (1.1% PFA solution for 10 min) followed by a 2000 rpm cytospin step for 5 min. In this latter case, the glass slides with the cytospun EVs were submerged in 1× PBS overnight before the fluorescent staining on the following day.

### 2.11 Fluorescence Staining of CTCs and EVs

For the fluorescent staining of the CTCs and EVs, three different strategies were utilised in this study: direct staining (DS), primary + secondary staining (PSS) and TSA staining (TSA). The staining was based on a protocol that was developed in the lab for TSA staining and then adapted for the other two strategies (DS and PSS). To minimise variability across the three methods, all the preparatory steps, washing steps, incubation times of the washing solutions/antibodies and the concentrations of the antibodies were kept constant. The only difference between the three techniques was the type of antibodies used for the staining, which included only a fluorescently labelled primary antibody for the DS method, an untagged primary antibody + a fluorescently labelled secondary antibody for the PSS method, and an untagged primary antibody + an HRP‐conjugated secondary antibody + a fluorescent TSA probe for the TSA approach.

The general complete staining protocol, developed and used in this study for the TSA staining of CTCs and EVs, is described in Supporting Information. Table 1 summarises the main steps, highlighting the most crucial parts of the protocol and the main differences between the staining of CTCs and EVs.

**TABLE 1:** Main steps of the TSA protocols developed for the staining of CTCs and EVs. A tick indicates the presence of the step for that sample, while a cross indicates its absence.

Protocol step	CTCs	EVs
Washing and pre‐block with Fab fragment IgGs	ü	ü
Washing and block with blocking solution	ü	ü
First cycle primary antibody incubation (in TNB)	ü	ü
First cycle secondary antibody incubation (in TNB)	ü	ü
First TSA probe incubation (in amplification diluent)	ü	ü
Quenching with 3% H_2_O_2_ solution	û	ü
Washing and block with blocking solution	ü	ü
Second cycle primary antibody incubation (in TNB)	ü	ü
Second cycle secondary antibody incubation (in TNB)	ü	ü
Second TSA probe incubation (in amplification diluent)	ü	ü
Washing and block with blocking solution	ü	û
Incubation with DAPI (in TNB)	ü	û
Final washing	ü	ü
Drying of the final solution	ü	û
Mounting slide with coverslip and sealing	ü	ü

For DS and PSS staining, the TSA protocol was slightly adjusted as described in the Supporting Information. The compositions of the TNT washing buffer and the TNB diluent solution, the concentrations of the staining antibodies and the DAPI, and the matching of the various primary‐secondary antibodies are also reported in the Supporting Information.

### 2.12 Imaging of GBM CTCs and EVs

The stained GBM cells/CTCs were imaged using a Vectra3 (v. 3.0.2) automated quantitative widefield microscope from Akoya Biosciences (40× air, NA = 0.95) and a LMS 710 confocal microscope from Zeiss. For CTC enumeration in patient samples, an age‐matched healthy control sample was imaged alongside every batch of three patient samples (stained at the same time), and the resulting images were analysed by two blinded reviewers. The GBM EVs were only imaged under the LMS 710 Zeiss confocal microscope due to the need for a high‐power oil immersion lens (63× oil, NA = 1.4). For the widefield imaging of the cells, the following acquisition times were used: 110 ms for DAPI, 750 ms for AF488 and 100 ms for AF594. For confocal imaging, the acquisition parameters were adjusted according to each sample (cells or EVs) and fluorophores, keeping them constant across the different staining methods for the comparisons. The confocal images of both cells and EVs were acquired using the smart setup option of the Zeiss ZEN black software, which guarantees minimal to no crosstalk between the different channels and, therefore, fluorophores.

### 2.13 Image Analysis of GBM CTCs and EVs

For cells/CTCs, the acquired images were spectrally unmixed using a built‐in library in the software *inForm*. This process eliminated possible cross‐talks between the acquired fluorescent channels. *HALO* software (Indica Labs, HighPlex FL v.4.1.3 module) was subsequently used to process the unmixed images. In details, a user‐trained classifier was applied to distinguish CTCs from WBCs, based on representative examples. Thereafter, a segmentation algorithm was used to detect and quantify marker expression. The analysis parameters included nuclear detection based on DAPI with a nuclear contrast threshold of 0.5 and intensity >10; membrane detection based on AF488 or AF594, with cytoplasmic positivity thresholds adjusted according to the experimental dye and a cytoplasmic completeness threshold >30% to minimise inclusion of potential staining artifacts. The output was a text file reporting the FL intensity values and sizes of all positively identified cells (above thresholds), which formed the basis for downstream analysis.

The acquired EV images were processed using one or a combination of the following software: *Zeiss ZEN Blue* and *Fiji*. Specifically, *ZEN Blue* software was used to calculate the FL intensity values of single EVs, by using the following workflow: automatic segmentation based on intensity histogram, intensity threshold set to detect fewer than 10 particles in control (EV‐free) substrates, morphological separation of objects and enabling the ‘Fill Holes’ function. *Fiji* software was primarily used for rapid EV counting/FOV when intensity values were not required and to determine the LOD in terms of the minimum number of detectable proteins/EV. For the first analysis, the *Process: Find Maxima* function was used, with a noise tolerance yielding fewer than 10 particles in control substrates. For the LOD experiments, the following sequence was applied: *Image: Adjust Threshold* function with a threshold yielding fewer than 10 particles in controls, followed by *Analyse: Analyse particles* function (minimum particle size of 10 pixels for EVs). The output of each analysis was a Result file containing information about the FL intensity values and size of each EV that was detected as positive (above thresholds), which were used in the analyses presented in this study.

## 3 Results

### 3.1 Comparison and Validation of TSA Staining Method on Single Circulating Tumour Cells

**FIGURE 1:**
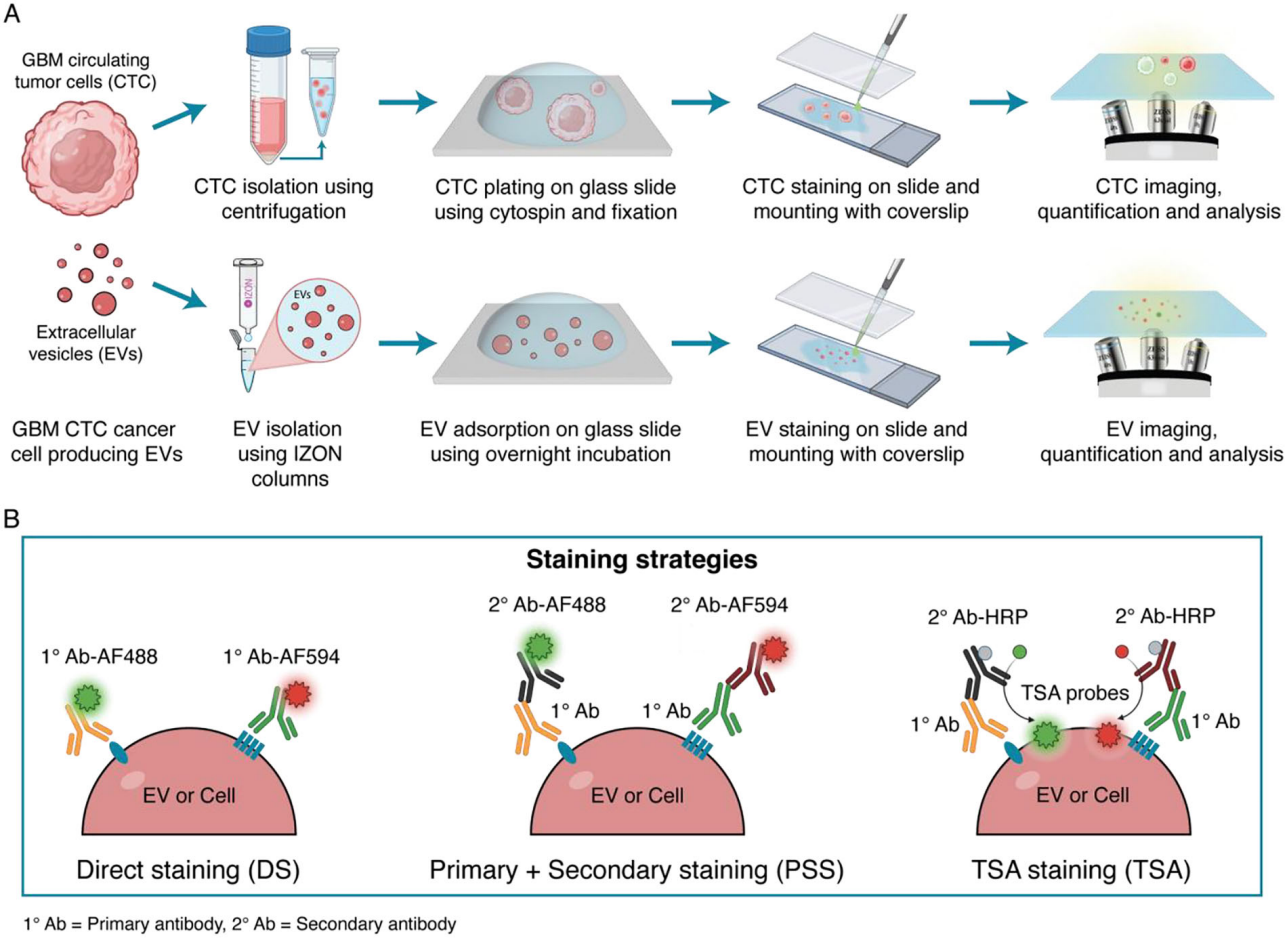
(A) Schematics of the process, from GBM cell culture to cell/CTC and EV isolations, immobilizations onto the glass substrates, fluorescent staining and imaging. (B) Schematics of the three different fluorescent staining strategies utilised in this study. From left to right: direct staining (DS) using fluorescently labelled primary antibodies; primary + secondary staining (PSS) using unconjugated primary antibodies followed by fluorescently labelled secondary antibodies; TSA staining (TSA) using unconjugated primary antibodies followed by HRP‐conjugated secondary antibodies followed by a fluorescent tyramide probe (usually AlexaFluor, such as AF488 or AF594) reacting with the HRP of the secondary antibody.

To validate the developed TSA staining technique, EVs and CTCs were obtained from two GBM cell lines established at Mass General Hospital, here named GBM1 and GBM2. As depicted in Figure 1A and described in Materials and Methods, the cells were collected using a 300 × *g* centrifugation step for 5 min followed by two resuspensions in PBS. The EVs were isolated using a combination of centrifugation (300 × *g* for 5 min + 2000 × *g* for 10 min), concentration (Amicon Ultra 15 mL Filters) and SEC (Izon qEV columns, 2nd generation). Following isolation, the cells were immobilised onto TB380 glass slides using cytospin and paraformaldehyde (PFA) fixation, while the EVs were adsorbed onto the glass slides using overnight incubation, unless otherwise specified.

The immobilised cells and/or EVs were then stained at a single particle level using the developed TSA staining technique, or a direct staining (DS) or a primary + secondary staining (PSS) for comparisons. Figure 1B shows a schematic of these three fluorescent strategies, while the detailed descriptions are provided in the Materials and Methods section and in Supporting Information. Briefly, while the DS and PSS methods utilise fluorescently labelled antibodies, the TSA strategy employs an unconjugated primary antibody to target the protein of interest, followed by an HRP‐conjugated secondary antibody. The fluorescent signal is then generated using an unreactive TSA probe, which is enzymatically converted into a reactive fluorophore by the HRP linked to the secondary antibody. Following staining, the slides with the fluorescently labelled cells or EVs were covered with a 170 µm coverslip (using either PBS or mounting media in between the two glass surfaces) and scanned under a fluorescent microscope (see Materials and Methods for details).

**FIGURE 2:**
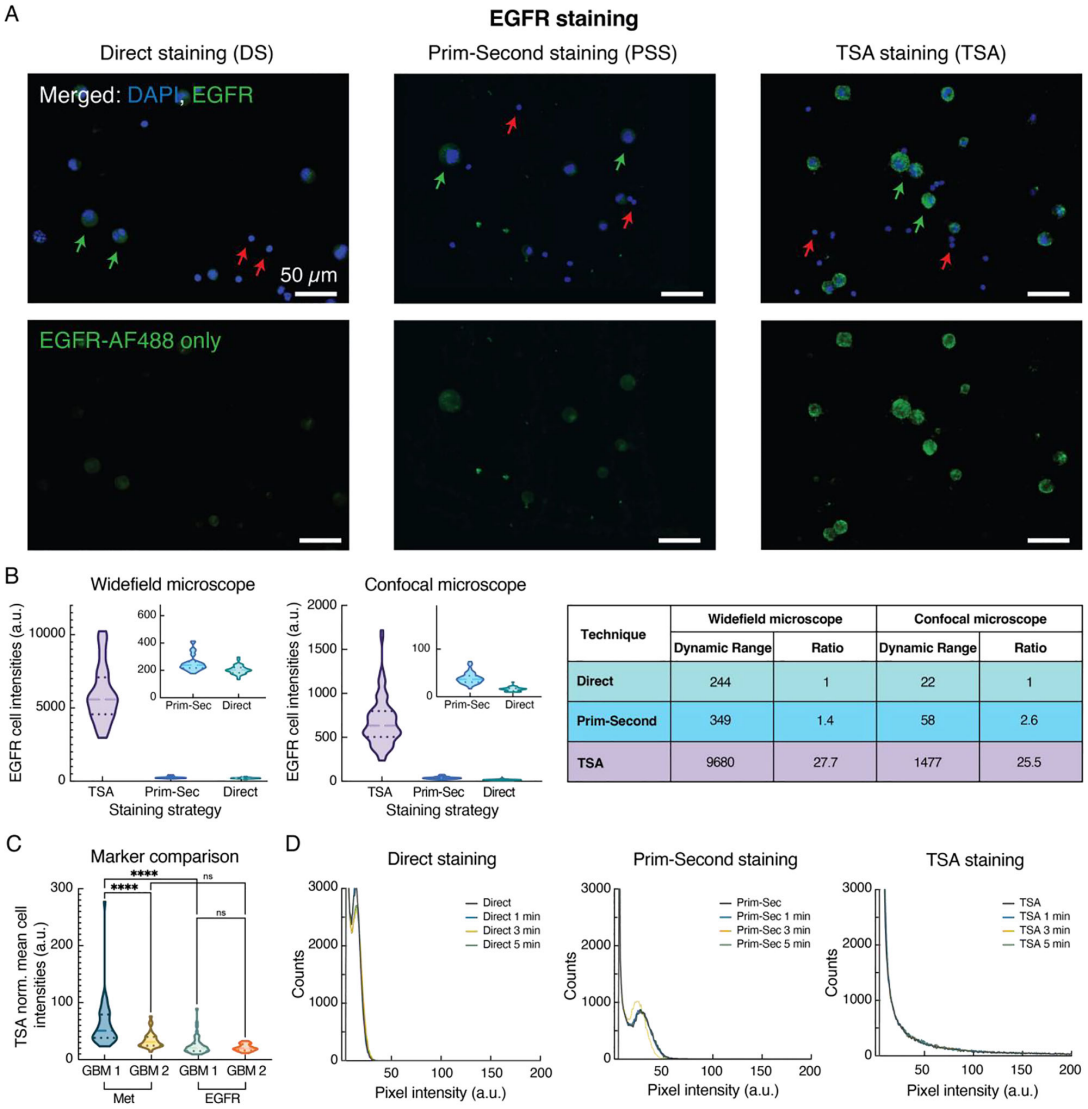
(A) Representative images showing staining of EGFR on GBM cells using direct, primary + secondary and TSA staining. Top panel: merged images of DAPI and EGFR‐AF488 channels. Bottom panel: split view of the EGFR‐AF488 green channel. Some of the GBM cells are highlighted with green arrows, while some of the WBC with red arrows. (B) Comparison of the distributions of EGFR intensities on single GBM cells for the three staining strategies, using two different fluorescence microscopes, widefield and confocal. The intensities were calculated considering five fields of view (FOV) for each staining strategy. The table on the right‐hand side shows the dynamic ranges and their ratios for the different staining methods. The dynamic ranges were calculated as the difference between the highest and lowest intensity values of the truncated violin plots presented in the figure. (C) Normalised mean cell intensity distributions, calculated for the TSA method only, for the two cell lines (GBM1 and GBM2) and two markers (EGFR and Met) analysed. (D) Time dependence of EGFR signal over a 5‐min period for the three staining strategies. The intensity of each pixel positive for EGFR (x‐axis, measured in arbitrary units, a.u.) was considered for this analysis. The y‐axis represents the number of EGFR‐positive pixels having a specific intensity. The coverslips were mounted on top of the glass slides with the CTCs using only PBS to avoid signal stabilization created by the use of a mounting media.

The TSA technique was first validated and compared with the other two techniques (DS and PSS) using parent GBM circulating tumour cells and white blood cells (WBC). To show the specificity of the technique and amplification of the fluorescent signals, GBM cells isolated from one of the cell lines (GBM1) were first plated on a slide in a 1:1 ratio together with WBC, isolated from a healthy donor using density gradient (BD Vacutainer CPT Mononuclear Cell Preparation Tubes, cat. # 362760, BD Biosciences). The plated cells were stained using DS, PSS and TSA staining. Epidermal growth factor receptor (EGFR) was used as a target marker due to its known over‐expression on the surface of these GBM cells (Table S1) and was targeted using either a green AF488‐labelled antibody or an AF488‐tyramide probe (TSA‐AF488). As visible in Figure 2A, EGFR green, fluorescent signals were only detected on the GBM cells (bigger cells with bigger DAPI nuclei, some pointed with green arrows) and not on the WBC (smaller cells with smaller DAPI, some pointed with red arrows), indicating specificity of the three techniques, including the developed TSA method. A split view of the green EGFR channels (Figure 2A, bottom images) shows minimal to no cross‐reactivity with the WBC immobilised on the same slides. Moreover, the images clearly suggested stronger EGFR fluorescent signals for the TSA technique as compared to DS and PSS (Figure 2A,B).

When analysing the intensities of EGFR, calculated as integrated intensities over the whole cell surface, on single GBM cells (Figure 2B), the results showed a significantly larger distribution of the TSA signals as compared to the other two strategies (*p* < 0.001 for both TSA‐PSS and TSA‐DS). This trend was confirmed by using two separate imaging instruments, a Vectra3 widefield microscope and a Zeiss LSM 710 confocal microscope, and by comparing the dynamic ranges of all the methods and systems. As presented in the table in Figure 2B, the ratios between the dynamic ranges (calculated as PSS to DS and TSA to PSS) were similar across the two imaging systems (1.4 and 2.6 for PSS to DS, and 27.7 and 25.5 for TSA to PSS), suggesting a consistency of the results and of the fluorescence amplification factors.

Once validated on one cell line, the TSA technique was applied on both GBM cell lines and tested for two separate surface proteins that are usually expressed on GBM cells, EGFR and Met (Guo et al. 2017; Joo et al. 2012; Jun et al. 2012; Sullivan et al. 2014). For this analysis and comparison, the normalised mean intensity values were considered. Such normalised values were derived by dividing the total cell integrated intensity by its two‐dimensional area and acquisition time, aiming to compensate for the variations in the cell dimensions and image acquisition times. This approach was chosen to address the differing expression levels of the analysed markers on the two cell lines, which necessitated different acquisition times for optimal data collection. As presented in Figure 2C, both cell lines expressed the analysed markers with a significant overexpression of Met on GBM1 cells as compared to GBM2 CTCs (*p* < 0.0001), and compared to EGFR for GBM1 (*p* < 0.0001). Met was slightly more expressed (mean intensity = 33.9 a.u.) than EGFR (mean intensity = 21.1 a.u.) on GBM2 CTCs, but the overall difference was not statistically significant (*p* = 0.35). No significant difference in the EGFR mean cell intensity was detected between GBM1 and GBM2 (*p* = 0.96), even though GBM1 cells showed an increased dynamic range of the FL signals. Additionally, a larger number of GBM1 cells expressed EGFR in each field of view (FOV) as compared to GBM2 CTCs (data not shown). Finally, the stability of the EGFR fluorescent signals (using both PBS and mounting media in between the glass and coverslip surfaces) for the three staining strategies over a period of 5 min was investigated. For these experiments, the substrates with the immobilised CTCs that were stained for EGFR were constantly excited with a laser for 5 min, and snapshot images were captured at time 0 and after 1, 3 and 5 min, respectively. As shown in Figures 2D and S1, the fluorescent EGFR signals were quite stable for the whole duration of the laser excitations, independently of the staining method used. Therefore, even the lowest signals detected in the case of DS and PSS were stable for at least 5 min. Moreover, this trend was valid whether PBS (Figure 2D) or mounting media (Figure S1) were used to mount the glass substrate with a coverslip and as an imaging media.

Following this initial validation with a single staining colour, the possibility of using the developed TSA protocol for multiplexed staining of single cells was investigated. For this purpose, a two‐plex TSA staining was initially used to distinguish between different cell types, specifically GBM CTCs and WBCs, and then used to show the possibility of co‐localizing multiple markers on the same tumour cell.

To achieve the first goal, GBM2 CTCs were spiked in healthy WBC samples and stained using a combination of markers that are (over)expressed and/or more specific for glioblastomas, according to the literature (Grzmil et al. 2011; Guo et al. 2017; Joo et al. 2012; Jun et al. 2012; Tchoghandjian et al. 2010; Verhaak et al. 2010) and one of our previous studies (Sullivan et al. 2014). These markers, including Sox2, Tubulin, EGFR, A2B5 and Met, were combined in a so‐called STEAM cocktail and stained in green using a TSA‐AF488 probe. For the WBC identification, a combination of CD11c and CD45 tagged in red with a TSA‐AF594 probe was used for staining. DAPI was used to stain and identify the cell nuclei, making the staining tri‐plex. However, since its staining did not include a TSA probe, herein we refer to our TSA protocol as a two‐plex staining.

**FIGURE 3:**
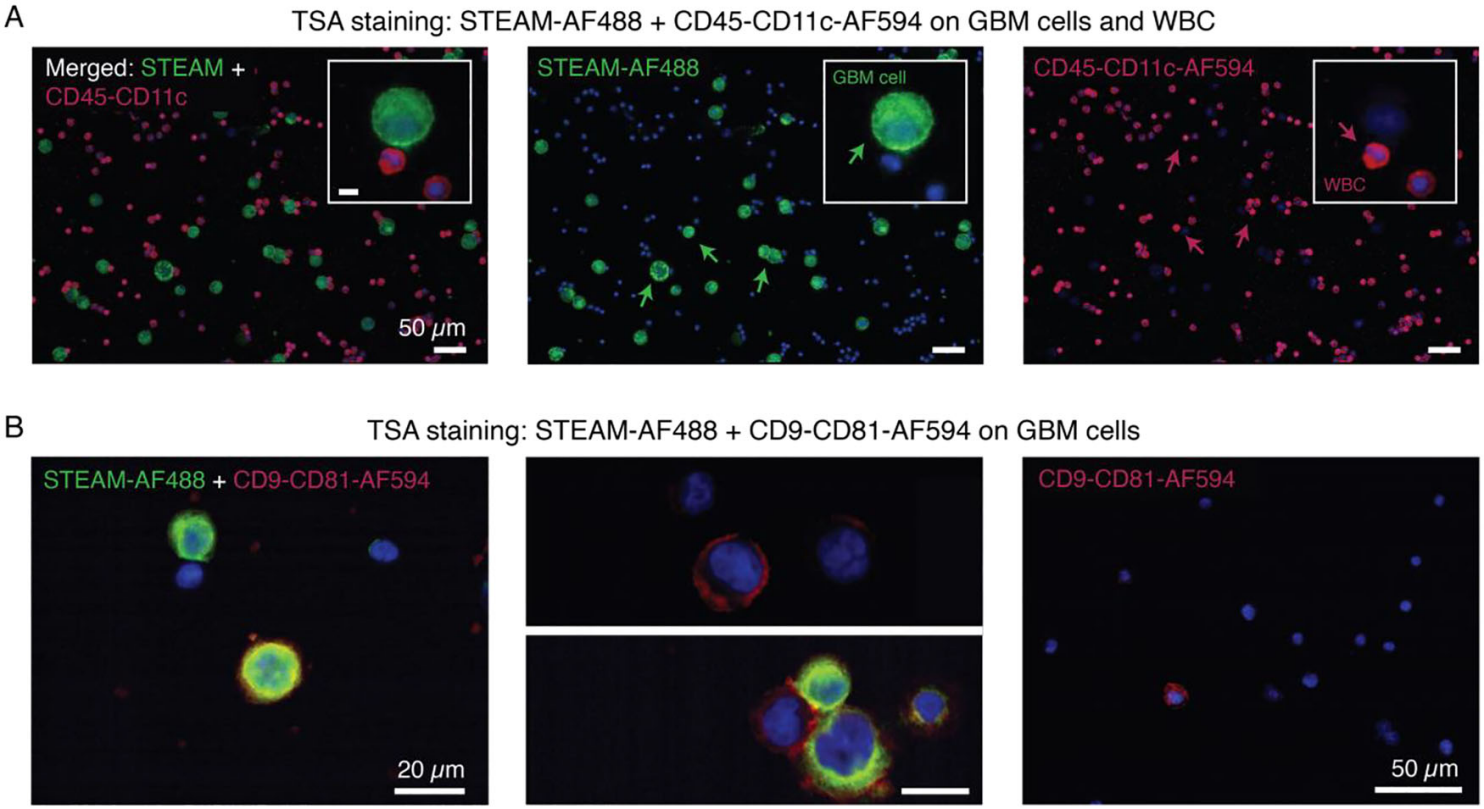
Representative images showing validation of the multiplexed TSA staining technique on cells. (A) GBM2 CTC cells spiked in healthy WBC samples and stained in green colour (with TSA‐AF488) for a cocktail of antibodies specific to GBM CTCs (STEAM) and in red colour (with TSA‐AF594) for CD45‐CD11c, two markers for WBC. From left to right: merged image of the STEAM‐AF488 and CD45‐CD11c‐AF594 channels; split view of the STEAM‐AF488 green channel; and split view of the CD45‐CD11c‐AF594 red channel. Blue staining of the nuclei corresponds to DAPI for all images. Scale bar = 50 µm for all images. Insets: zoom‐in on one GBM CTC (green arrow, inset in middle image) with two WBCs close by (red arrow, inset in right image). Scale bar of inset = 10 µm. (B) Two‐plex staining of GBM2 CTCs only using STEAM‐AF488 and a cocktail CD9‐CD81‐AF594. Many cells are only positive for STEAM‐AF488 (green colour) or CD9‐CD81‐AF594 (red colour). Some cells are double positive for both marker combinations (green + red colours). Scale bar = 20 µm for images in the middle. Not all cancer cells seem to express CD9‐CD81 on their surface.

As visible in Figure 3A, green signals corresponding to the STEAM cocktail were exclusively detected on GBM CTCs, while red signals were specific to WBCs. These findings aligned with the outcomes of our previous study (Sullivan et al. 2014), showing successful differentiation between GBM CTCs and WBC using these two antibody cocktails. However, while our previous work achieved these conclusions through direct fluorescent staining of the markers, this study reaffirms the same outcomes using the developed TSA protocol. When trying to perform multiplexed staining on the same cell type (GBM2 CTCs), the STEAM‐AF488 antibody cocktail was combined with a cocktail of CD9‐CD81 antibodies tagged in red using a TSA‐AF594 probe (Figure 3B). CD9 and CD81 are tetraspanin proteins that are commonly enriched on the surface of EVs (Théry et al. 2018). In this case, we investigated their potential presence on the surface of the parent GBM2 CTCs. As presented in Figure 3B, the two‐staining colours were detected on GBM CTCs, with most cells showing single positivity for either STEAM or CD9‐CD81 markers. Moreover, as visible in the image on the right of Figure 3B, when staining GBM2 CTCs for CD9‐CD81 only (no STEAM), not many circulating tumour cells seemed to express CD9 and CD81 on their surface, as compared to STEAM (Figure 3A, middle).

Here, we would like to highlight that the use of a quenching buffer between the first and second staining colours was not needed, as the signals were specific to the CTCs and WBCs even in the absence of this buffer. Moreover, as some of the targeted markers were intracellular (Sox2, Tubulin), a small amount of Triton X‐100 (0.3%) was included in the staining protocol, specifically in the TNB antibody diluent buffer, for permeabilization. However, the results showed that the presence of Triton X‐100 did not cause cell break or lysis, as shown by the intact aspect of the imaged cells (Figure 3). These two aspects need to have particular attention when staining EVs and will be further discussed later.

Overall, the two‐plex staining results obtained with GBM CTCs and WBCs suggested negligible crosstalk between the two‐staining colours, whether they are used to stain two different types of cells or two marker combinations on the same cell, highlighting the possibility of performing multiplexed staining on cells using the developed TSA technique.

### 3.2 Characterization of EVs and Optimization of Their Capture Onto Glass Substrates

As previously demonstrated with cells, compared with traditional staining strategies using fluorescently labelled primary or secondary antibodies, the developed and presented TSA technique shows noteworthy advantages, including (a) minimal non‐specific staining, (b) amplification of the fluorescent signals, (c) high signal dynamic ranges and (d) high signal stabilities over time. These features assume critical importance when staining single EVs, given the very small size (<400 nm) of most vesicles and the very low abundance of many markers/proteins of significance in cancer. The combination of these characteristics makes the staining of single vesicles extremely challenging, as the fluorescent signals are exceedingly weak or tend to disappear even before image acquisition.

Prior to validating and applying the developed TSA staining technique to single vesicles, some preliminary characterization experiments were performed to ensure proper EV isolation from the GBM parent cells. Collection and capture of EVs are indeed more challenging than cells due to the small size of these vesicles, their heterogeneity and their overlap in size and density with other particles present in body fluids or cell media, such as protein aggregates or lipoproteins (Chou et al. 2024).

**FIGURE 4:**
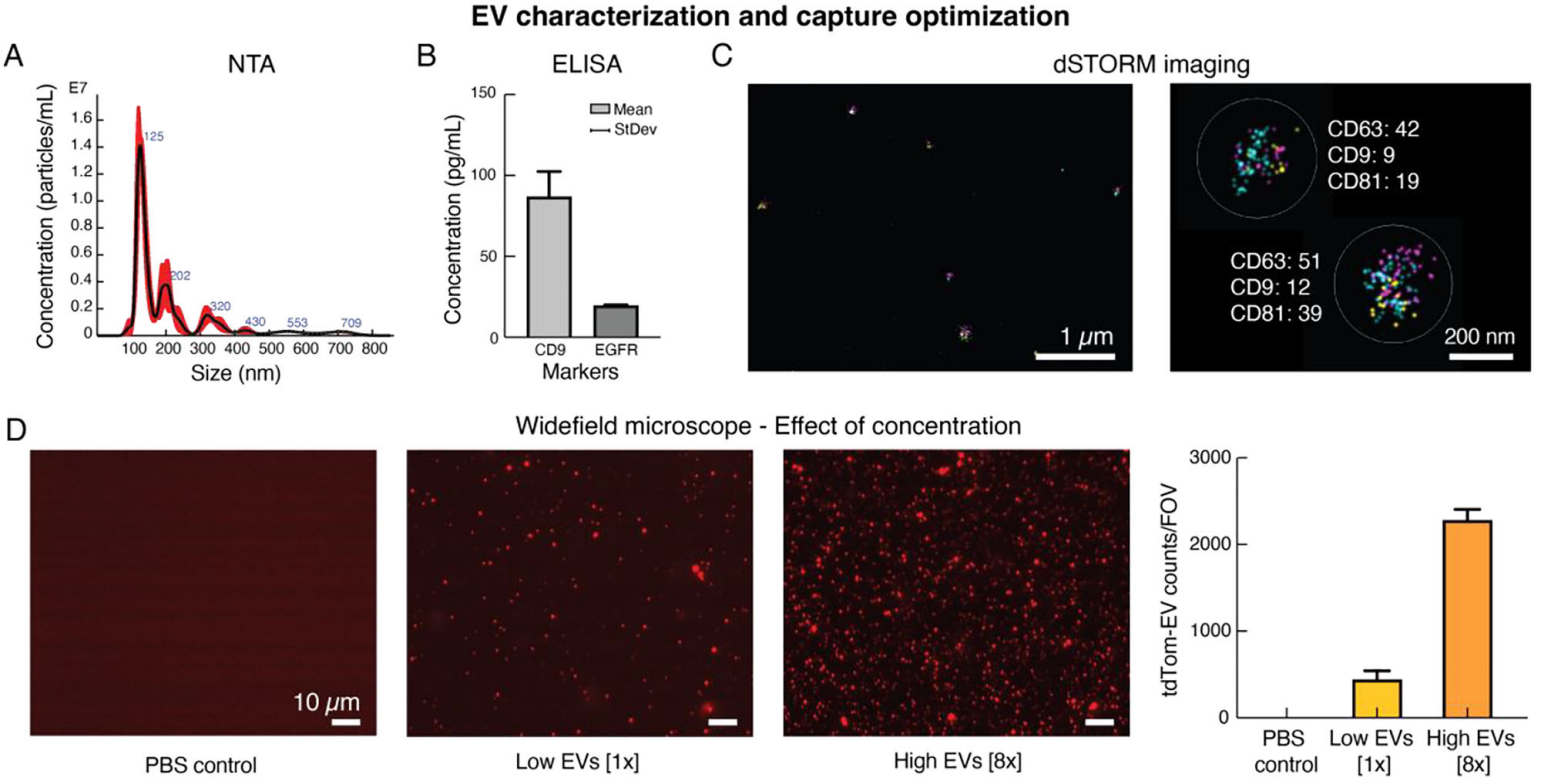
(A) Characterization of the size distribution of the EVs isolated from GBM2 CTCs by Izon SEC using NTA. Measured total concentration: 0.9 × 10^9^ particles/mL. (B) Characterization of the GBM2 EV protein content (CD9 and EGFR) using ELISA. (C) Characterization of the EV tetraspanin expression and distribution using ONI dSTORM imaging system. CD9‐AF488 (yellow dots), CD63‐AF555 (blue dots) and CD81‐AF594 (pink dots) targeted and analysed. Right hand‐side image: zoom‐in on two EVs co‐expressing the three analysed tetraspanins. The numbers reported beside each tetraspanin represent the number of dots detected on each vesicle and are proportional to the amount/expression of that specific tetraspanin on the EV surface. (D) Validation of the EV capture onto a glass substrate. EVs labelled with tdTomato fluorophore were adsorbed overnight on a TB380 glass slide and imaged the day after, after several washing steps. Two different concentrations (Low EVs = [1×] = 3 × 10^7^ particles/mL and High EVs = [8×] = 24 × 10^7^ particles/mL) tested, and PSB used as a control. EV counts obtained from four FOVs and plotted on the right as mean ± SD. Images obtained with the 100× oil immersion lens (NA 1.45) of a widefield microscope (Nikon 90i) equipped with a cooled CCD camera (Andor Clara DR‐2519) and a 1.6× optical coupler (Nikon Digital Imaging Head).

Following isolation using Izon SEC columns, the size and concentration of the EVs were first characterised using NTA (Figure 4A), their protein content was verified by ELISA (Figure 4B), Western Blot (Figure S2) and dSTORM imaging (Figure 4C), while their morphology was evaluated by transmission electron microscopy (TEM, Figure S2). As presented, the isolated samples contained particles mostly in the small EV (sEV) size range (<400 nm, Figure 4A) with concentrations ranging from 0.9 × 10^9^ to 4 × 10^9^ particles/mL. The particles showed several markers usually present on the EVs, such as CD9, CD63, CD81, Alix and HSP70 (Figure 4B, C‐S2A), as well as the absence of cellular contamination via Calnexin (Figure S2A). TEM images revealed the presence of lipid‐bilayered vesicles within the expected EV size range, without major contaminants, supporting the purity of the isolated vesicles (Figure S2B). Collectively, these characterization experiments confirmed the presence of EVs in the isolated samples.

For antibody staining experiments, it is essential that the vesicles are strongly and stably immobilised onto a substrate such that they can withstand multiple washing cycles. To determine the best capture strategy, following isolation and characterization, the EVs were immobilised on top of different substrates and counted after various plating conditions, concentrations and washing cycles. For the sake of simplicity, intrinsically labelled EVs tagged with tandem dimer Tomato (tdTomato) (Lai et al. 2015), here referred to as tdTom‐EVs, were isolated from tdTomato expressing GBM cells (see Materials and Methods for details) and used for these experiments. Specifically, the vesicles were immobilised onto two different coated glass slides: a Poly‐L‐lysine (PLL) and a TB380 slide. Further, we evaluated each surface by plating the EVs using overnight adsorption or using centrifugal forces (e.g., cytospin). Two different EV concentrations, Low EVs = [1×] and High EVs = [8×], were used for all the plating conditions and the vesicles were counted after multiple washing steps (5–10) with PBS. As shown in Figures 4D and S3, the best EV capture and retention was obtained on top of the TB380 slide with overnight adsorption of the vesicles. This strategy ensured the highest average number of captured EVs/FOV (∼2300 for High EVs, Figure S3B), the most uniform distribution of the vesicles over the substrates, and the best proportion between the two different EV concentrations analysed (Figure 4D, right plot, and Figure S3B: EV ratios [8×]/[1×] = 5.5 for TB380 and [8×]/[1×] = 1.8 for PLL). Importantly, the FL intensity distribution of the particles detected as EVs (Figure S3C) remained consistent across the two different EV concentrations, with no shift toward higher intensity values at the higher concentration tested, High EVs [8×]. This finding indicated that single EVs were primarily immobilised and analysed. Moreover, the EVs remained stably immobilised on top of the substrates for at least 3 days (Figure S4), upon storage of the slide at 4°C. Considering these results, TB380 slides were used as EV substrates for the subsequent experiments using the developed TSA technique.

### 3.3 Validation and Application of the Developed TSA Staining on Single EVs

Following the initial validation of the staining technique on cells and the optimization of the EV capture to the substrates, the applicability of the developed TSA technique for the staining of single EVs was systematically investigated and confirmed. Specifically, the vesicles isolated from the GBM2 CTC line were stained at a single particle level using the developed signal amplification approach as well as the two conventional methods of direct staining (DS) and primary + secondary staining (PSS). Since these vesicles lacked intrinsic fluorescence, the surface protein CD63 was selected as a marker and stained using fluorescently labelled antibodies and/or TSA probes. For the staining of these EVs, the same protocols used for the GBM CTCs (see Materials and Methods and Supporting Information) were followed, with the exceptions that (1) the DAPI step was skipped, (2) the vesicles were not dried at the end of the protocol, prior to the coverslip mounting, and that (3) a lower concentration of Triton X‐100 (0.01% as compared to 0.3% for cells) was included in the diluent buffers. The latter adjustment was based on the fact that lipid membrane‐enclosed vesicles, such as EVs, have been shown to be sensitive to detergent lysis (del Pozo‐Acebo et al. 2021; Osteikoetxea et al. 2015; Romanò and Cinti 2023) and, therefore, necessitate a meticulous choice of detergent type and concentration to preserve their structural integrity. Since a study from Osteikoetxea et al. (2015) demonstrated that a 0.01% Triton X‐100 concentration guaranteed the best preservation of both exosomes and microvesicles, this specific detergent concentration was adopted and integrated into our staining protocol of the EVs.

**FIGURE 5:**
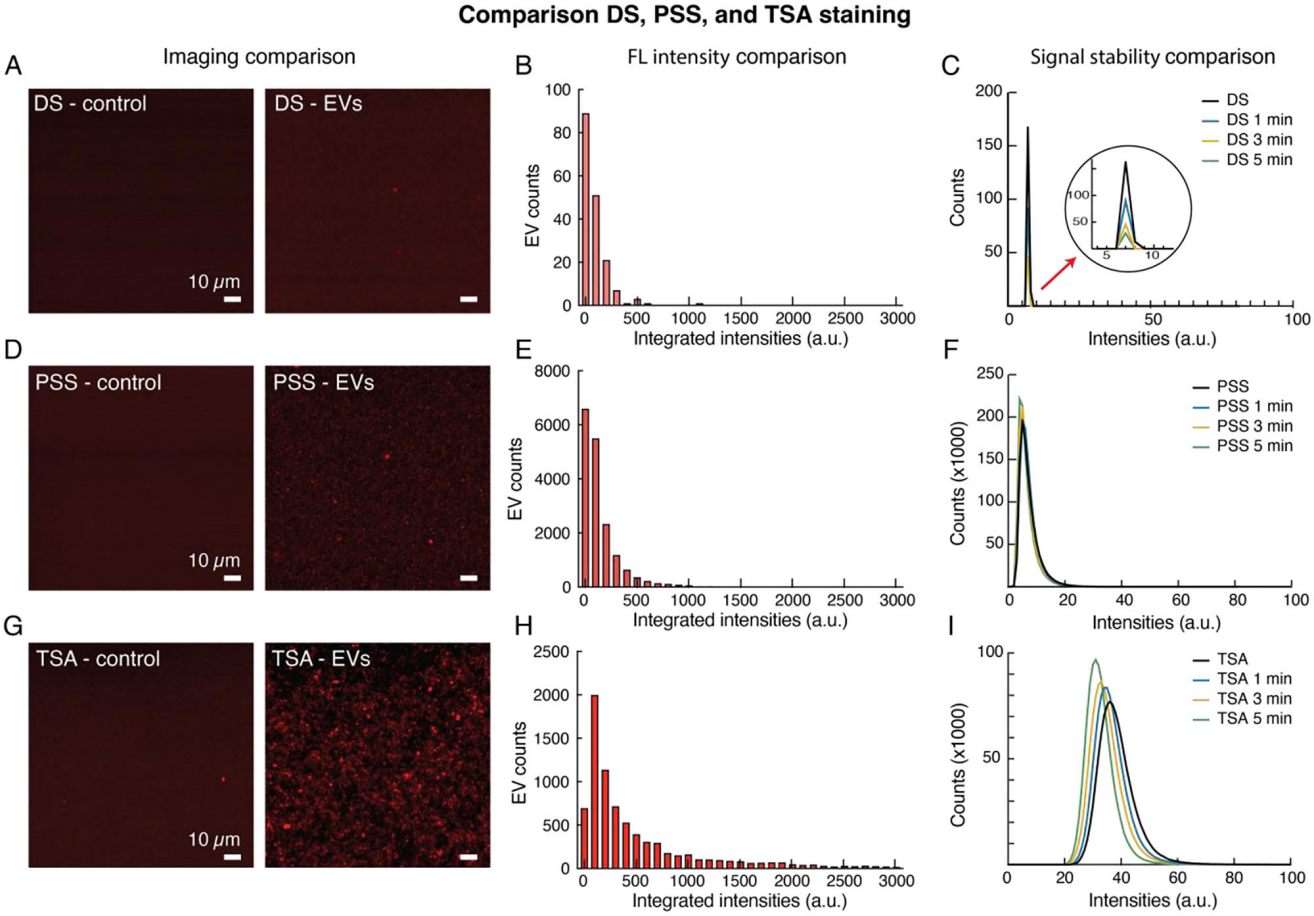
Comparison between DS, PSS and the developed TSA staining protocol using GBM2 EVs. (A, D, G) Representative images of the EVs stained using the three different techniques along with the respective control substrates (no EVs, PBS only + CD63 antibodies). (B, E, H) Fluorescent intensities (plotted as integrated intensities over all the pixels of each single EV) and counts of single EVs stained with the three different techniques, calculated considering two FOVs per technique. (C, F, I) Profiles of the EV pixel intensities over a 5‐min period of continuous laser excitation for the three analysed techniques. The four curves correspond to the pixel intensity distributions of the snapshot images captured at time 0, and after 1, 3 and 5 min of laser excitations, respectively.

As presented in Figure 5A, D and G, all three methods showed specific staining of single vesicles as compared to their respective PBS controls (substrates without EVs, stained with fluorescent CD63 antibodies or TSA probes). However, the fluorescent signals obtained through the direct staining method were notably weaker than those obtained using the other two methods, illusorily showing a very small number of positive EVs per FOV (<100). Given that the plating concentration of the vesicles was the same across the three different substrates, such a low number of positive EVs, compared to the other two methods (where positive EVs per FOV was >2000), was attributed to the fact that the majority of the CD63 signals on the EVs were weaker, equal, or not significantly stronger than the background signals of the substrates, therefore concealing the vesicles. On the other hand, the TSA staining method showed the strongest CD63 intensities among all the techniques, once again demonstrating the desired amplification of the fluorescent signals. This trend in the fluorescent signals can be further observed, confirmed and quantified in the plots in Figure 5B, E and H, which show the distribution of the CD63 intensities on single vesicles for the three methods. As visible, the DS approach showed the weakest CD63 integrated intensities (average intensity = 95 a.u.) and lowest EV counts/FOV among the three methods, while the TSA approach showed the highest intensities (average intensity = 607 a.u., 6.4× higher than the DS method). Not surprisingly, the PSS method showed intermediate CD63 intensities (average intensity = 143 a.u., 4.2× lower than the TSA method), but higher EV counts/FOV (∼7500) than the TSA approach (∼4500).

When looking at the stability of the fluorescent signals over time (Figure 5C,F and I), a major difference compared with the staining of the GBM cells is that for the EVs, the continuous excitation with a laser drastically reduces the signals obtained with DS, even after a period of 1 min (Figure 5C). On the contrary, and similarly to the CTC staining, the signals are quite stable when using PSS (Figure 5F) and TSA (Figure 5I). However, the TSA approach notably shows the advantage of higher (∼3×) dynamic ranges of the signals (IntI = 7150 and PixI = 130) compared to the PSS method (IntI = 2200 and PixI = 48), as presented in Figures 5E,H and S5. As for CTC staining, these dynamic ranges were calculated as the differences between the highest and lowest intensity values of the integrated (IntI) and pixel (PixI) intensity distributions presented in Figure S5. These findings make the TSA method a more suitable technique for the staining of low abundant markers and/or their comparison across a cohort of different samples.

Overall, the results in Figures 5 and S3–S5 demonstrate the possibility of applying the developed TSA protocol for the specific staining of single EVs and highlight the advantages of amplified and more stable signal intensities and broader signal dynamic ranges of this technique as compared to conventional methods (DS and PSS).

### 3.4 Two‐Plex Staining of Single EVs Using the TSA Method

Following the validation of the developed TSA method on the EVs and the comparison of the technique with conventional IF staining approaches, the possibility of using TSA for multiplexed staining of single EVs was explored. To the best of our knowledge, this study is the first one investigating and demonstrating this possibility on single vesicles, as the few other reports using a TSA method on EVs (Chen et al. 2022; Nguyen et al. 2022; Reynolds et al. 2023) only demonstrated single‐plex staining. Moreover, in this work, the developed TSA method is used to target both generic and GBM‐specific EV markers.

For this set of experiments, EVs isolated from the GBM2 CTC line were used and their two‐plex staining using a green and a red TSA probe was investigated. The red tyramide probe, TSA‐AF594, was used to target the EV surface markers CD9 and CD81 through a cocktail of these two antibodies. This colour represented the ‘generic EV marker’. The choice of CD9 and CD81 was based on some preliminary staining experiments performed on the same GBM2 EVs (data not shown), which revealed that this tetraspanin combination tagged a higher number of EVs in each FOV than that obtained using CD63 as a target. For consistency with the staining performed on GBM2 CTCs, the green tyramide probe, TSA‐AF488, was used to target the possible presence of STEAM markers on the EVs. This colour represented the ‘GBM‐specific EV marker’ and was expected to identify a smaller proportion of EVs as compared to the tetraspanin staining. Here, we would like to mention that the matching of the green TSA probe with the STEAM cocktail and of the red probe with the CD9‐CD81 cocktail was arbitrary. The staining colours can be switched without any constraint.

**FIGURE 6:**
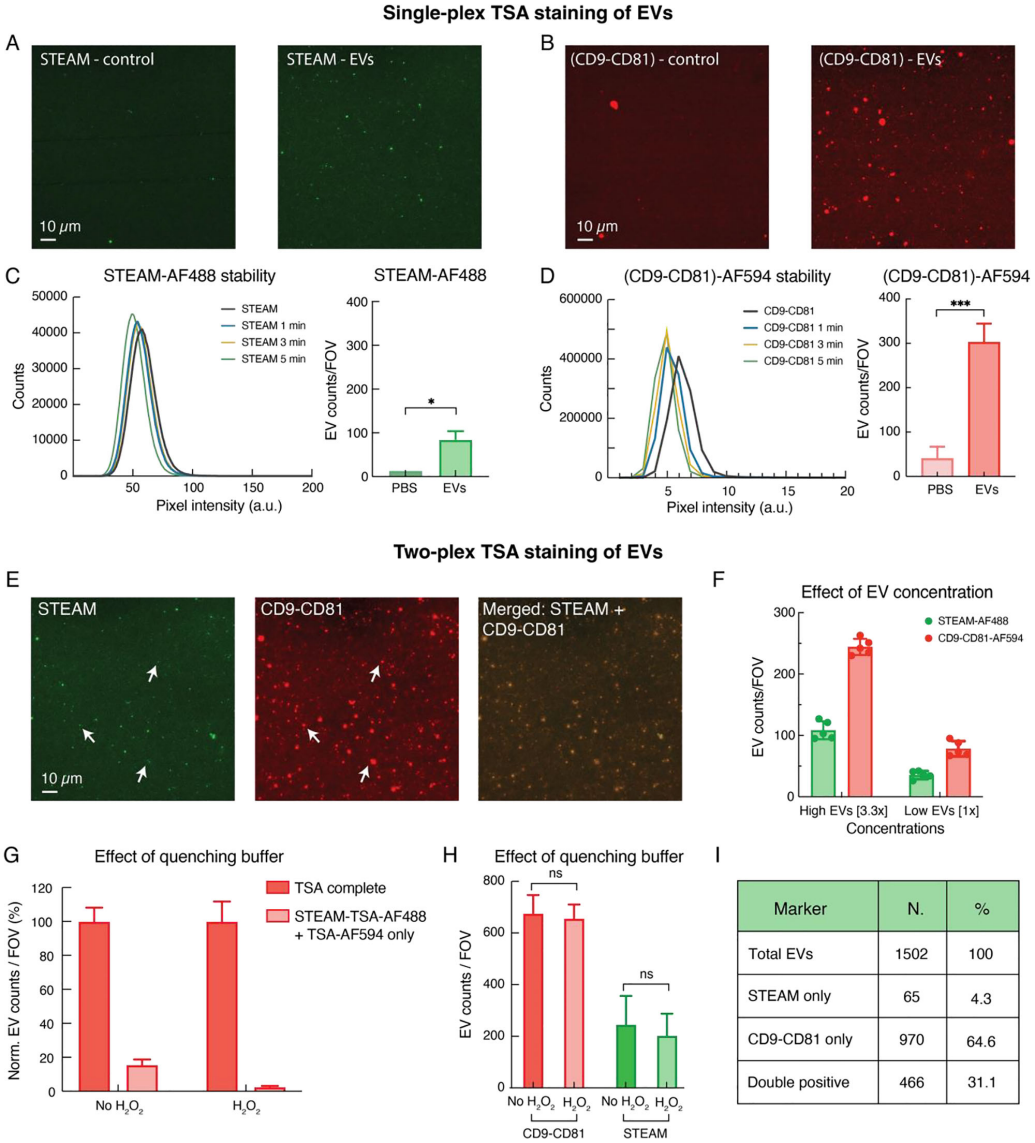
(A, B) Representative images of two substrates with EVs and two respective PBS control substrates, separately stained for STEAM, using TSA‐AF488, and CD9‐CD81, using TSA‐AF594. (C, D) Profiles of the EV pixel intensities over a 5‐min period of continuous excitation for both STEAM‐AF488 and CD9‐CD81‐AF594. The four curves correspond to the pixel intensity distributions of the snapshot images captured at time 0, and after 1, 3 and 5 min of laser excitations, respectively. Plots on the right show the number of particles detected per FOV on the EV substrates as compared to their respective PBS substrates. EV counts obtained from four FOVs and plotted as mean ± SD. (E) Representative image of a substrate with EVs that was stained for STEAM and CD9‐CD81 using the two‐plex staining protocol. The white arrows highlight some of the EVs that co‐expressed both marker combinations on their surface. (F) Number of EVs showing positivity for STEAM and CD9‐CD81 detected per FOV, for the two different EV concentrations analysed, [1×] and [3.3×]. EV counts obtained from five FOVs and plotted as mean ± SD. (G) Number of EVs, calculated as normalised EV counts (%), showing positivity for CD9‐CD81 in the presence (H_2_O_2_) and absence (no H_2_O_2_) of H_2_O_2_ quenching buffer for two staining conditions. TSA complete represents the number of CD9‐CD81 positive EVs detected when using the complete two‐plex TSA staining protocol. STEAM‐TSA‐AF488 + TSA‐AF594 only represents the number of CD9‐CD81 positive EVs detected when incubating the EVs with a TSA‐AF594 probe directly after the completion of the first TSA staining cycle with STEAM + TSA‐AF488 probe. In this case, no primary and secondary antibodies directed to CD9‐CD81 were used, but the TSA‐AF594 was introduced to test its cross‐reactivity with the HRP‐conjugated secondary antibodies used to target the STEAM markers. The normalised EV counts (%) were obtained by dividing the raw EV numbers obtained for the complete TSA protocols by the mean EV numbers, and by further multiplying x100. In this way, the number of CD9‐CD81 positive EVs detected with complete TSA staining corresponded to 100% for both presence and absence of H_2_O_2_ quenching buffer. (H) Number of EVs showing positivity for STEAM and CD9‐CD81 detected per FOV for an EV substrate stained with the complete two‐plex TSA protocol, including the H_2_O_2_ step (H_2_O_2_ bars), and two separate substrates having the same EV concentrations, one of which was only stained for STEAM with the TSA‐AF488 probe and the other one for CD9‐CD81 with the TSA‐AF594 probe. These two substrates are labelled with no H_2_O_2_. (I) Table showing the total number of EVs detected in 5 FOV and the numbers of EVs that were single positive (for STEAM or CD9‐CD81 only) and double positive (for both STEAM and CD9‐CD81). Percentages relative to the total number of EVs (100%) also included in the Table.

First, the two‐staining colours were tested separately to detect the possible presence and EV‐specificity of the TSA signals. The use of a cocktail of antibodies to target the GBM‐specific markers might challenge the staining by increasing NSB/background noise. However, the results in Figure 6A–D demonstrated the specificity of the signals toward the EVs. As clearly visible, for both colours/marker combinations, a significantly higher number of fluorescent spots were detected on the substrates with EVs as compared to their respective PBS control substrates without EVs. Figure 6C,D also show that both TSA fluorescent signals were quite stable over time, for at least 5 min of continuous laser excitations.

To estimate the limits of detection (LOD) of our TSA method, two additional sets of experiments were performed: one to evaluate the ability to detect single low‐abundance surface markers on the EVs and one to estimate the minimum number of proteins per EV that could be detected as positives. For the first set of experiments, two proteins from the STEAM cocktail, specifically EGFR and MET, were selected due to their higher GBM‐specificity and lower abundance compared to tetraspanins. These proteins were separately stained on individual EVs isolated from the two GBM cell lines by using the TSA protocol. The results presented in Figure S6 demonstrate the capability of our TSA method to detect these single low‐abundance markers on the EV surface. Moreover, they reveal significant differences in the protein expression levels between the two populations, with EGFR being more expressed on the GBM1 EVs while Met on the GBM2 EVs (Figure S6). To estimate the minimum number of detectable proteins per EV, control substrates without EVs incubated with HRP‐conjugated secondary antibodies followed by TSA probes were used. Specifically, diluted secondary antibodies (to ensure enough spatial separation) were immobilised on the substrates and exposed to the TSA probes (either green TSA‐AF488 or red TSA‐AF594) for 5 min, mimicking the substrates with EVs. As shown in Figure S7, both control substrates showed distinct fluorescent spots that could be attributed to single antibody events. For each TSA probe, the first peak of the distribution of the fluorescent signals was considered as the representative amplified signal of a single antibody. The minimum number of detectable proteins per EV was then estimated by dividing the first peak of the fluorescent distribution of the EV substrates by the single antibody event signal. As presented in Figure S7, the data from this analysis suggested that our method can potentially detect as few as two protein copies per EV for the surface markers examined in this study.

Thereafter, a two‐plex staining protocol was implemented to combine and test these two cocktail combinations on the same EV substrate. The developed approach first targeted the STEAM markers with TSA‐AF488 and then the CD9‐CD81 tetraspanins on the same EVs with TSA‐AF594. As shown in Figure 6E,F, fluorescent signals corresponding to both TSA probes could be detected on the EV substrates, suggesting the expression of the targeted markers by the vesicles and some colocalizations on single particles (highlighted by white arrows, Figure 6E). The specificity and applicability of the two‐plex staining was confirmed by the use of two different vesicle concentrations, Low EVs = [1×] and High EVs = [3.3×], which showed changes in the number of EVs detected per FOV proportional to the concentration changes (Figure 6F).

However, for the two‐plex TSA staining of EVs, we would like to highlight that a crucial step was introduced in this protocol as compared to the two‐plex protocol used for the GBM cells. In particular, to guarantee specific staining with both TSA probes and minimal cross‐reactivity, a quenching step consisting of the use of a 3% H_2_O_2_ solution (for 20 min) (Lee et al. 2018), was introduced after the completion of the STEAM staining cycle and before the start of the CD9‐CD81 cycle (see Materials and Methods for details). When performing the protocol without the use of this quenching buffer between these two staining cycles, some unspecific signal caused by the reaction of a still active endogenous HRP of the first secondary antibody with the second TSA‐AF594 probe was detected. This hypothesis was verified by testing two identical substrates, one of which was stained with the complete TSA protocol (STEAM with TSA‐AF488 and CD9‐CD81 with TSA‐AF594, named as ‘TSA complete’) while the other one with the first staining cycle (STEAM with TSA‐AF488) only, followed by incubation of the TSA‐AF594 probe (no CD9‐CD81 antibodies, named as ‘STEAM‐TSA‐AF488 + TSA‐AF594 only’). In both cases, no H_2_O_2_ buffer was utilised.

As shown in Figure 6G, in the absence of H_2_O_2_ quenching (left bars, No H_2_O_2_), about 20% of the total EVs detected with the TSA‐AF594 probe did not correspond to a real signal generated by the corresponding antibodies but to a signal that was caused by the reaction of the HRP of the secondary antibodies used to target STEAM with the TSA‐AF594 probe introduced afterward. On the contrary, when a 3% H_2_O_2_ quenching solution was used (right bars, H_2_O_2_), such cross‐reaction was negligible (only 3%), and all the signal detected in the red channel corresponded to specific binding of the CD9‐CD81 antibodies to the corresponding proteins on the EVs.

To further demonstrate that the introduction of the H_2_O_2_ quenching step did not negatively affect the staining of the vesicles, an EV substrate stained with the complete two‐plex TSA protocol, including the H_2_O_2_ step, was compared with two other substrates having the same EV concentrations, one of which was only stained for STEAM with the TSA‐AF488 probe and the other one for CD9‐CD81 with the TSA‐AF594 probe without any H_2_O_2_ step. For this analysis, the number of EVs detected per FOV was counted and compared across all substrates. As shown in Figure 6H, the number of EVs positive for STEAM and CD9‐CD81 remained similar for both staining conditions (*p* values not significant), indicating that the use of H_2_O_2_ did not modify the binding of the antibodies to their corresponding EV markers.

Overall, the results in Figure 6 show the feasibility of using the developed two‐plex TSA protocol on single EVs and the importance of utilizing a quenching step in between the different staining colours to guarantee a specific detection of the markers. Moreover, the data show that this type of staining offers the possibility to colocalise several markers or marker combinations on the same vesicles, potentially identifying vesicle subpopulations or correlations between the analysed parameters. For example, in the specific example presented in Figure 6, among all the EVs identified, most of the vesicles only expressed CD9‐CD81 (64.6%), while only 31% of the vesicles were double positive for STEAM and the analysed tetraspanins (Figure 6I). Moreover, there seemed to be a light correlation (*r* = 0.73) between the expression of the two groups of markers, with a linear correlation of the tetraspanin expression and the expression of STEAM (Figure S8A) on single EVs. Finally, the data showed that the EVs with the highest expression of the CD9‐CD81 tetraspanins were those that were also positive for STEAM (CD9‐CD81 double +, Figure S8B), while there was not a significant change in the expression levels of STEAM for the vesicles that were single positive for that marker cocktail or co‐expressed the analysed tetraspanins.

### 3.5 Application of the Developed TSA Protocol to GBM CTCs and EVs From Glioblastoma Patient Samples

**FIGURE 7:**
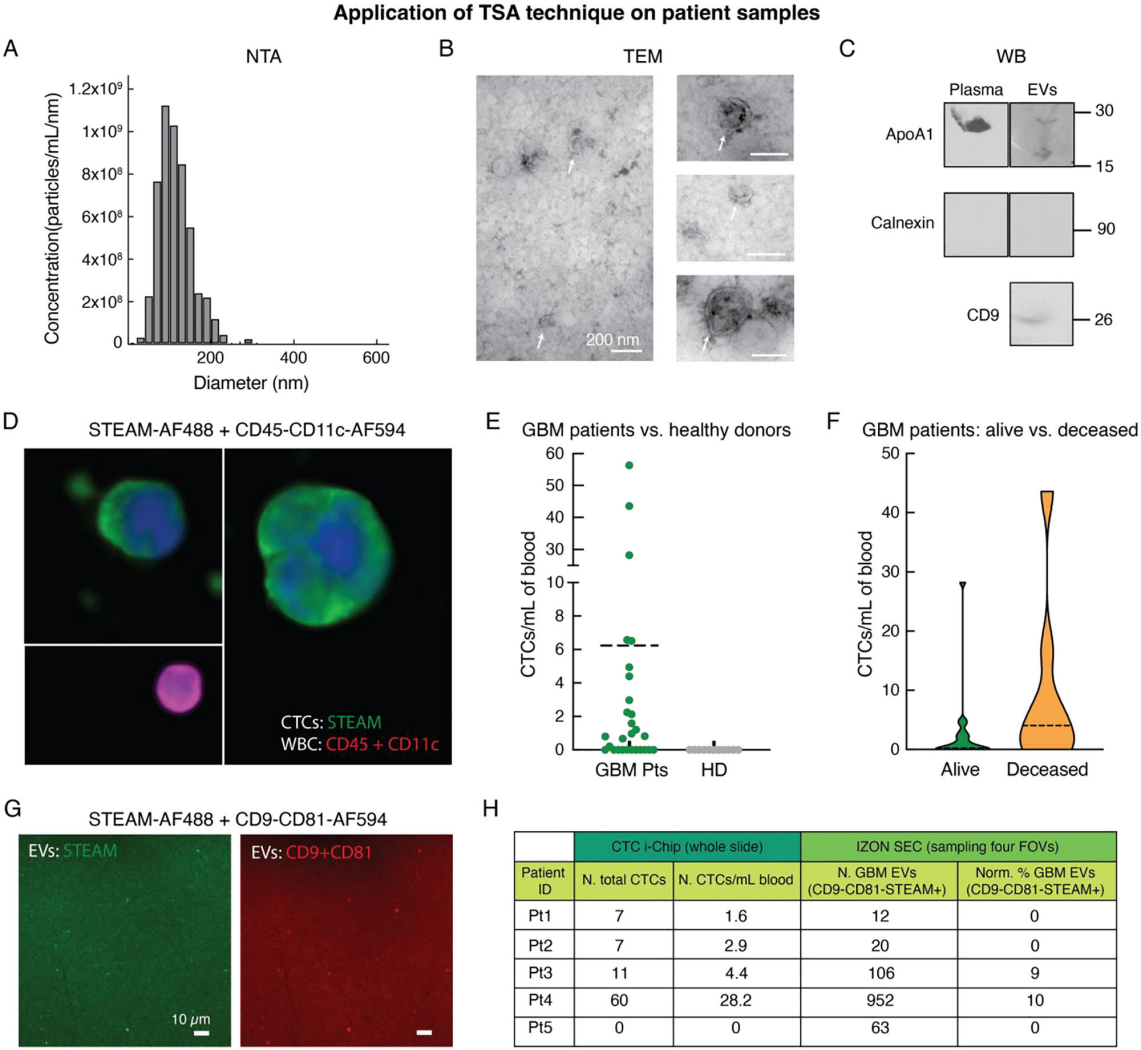
(A) Representative characterization of the particle size distribution of an EV sample isolated from a GBM patient plasma (Pt4) by Izon SEC using NTA (ViewSizer 3000, Horiba). Measured total concentration: 1.25 × 10^10^ particles/mL. (B) Representative TEM images of the plasma EVs isolated by SEC from GBM Pt4 patient, showing vesicles enclosed by a lipid bilayer in the size range of EVs (white arrows). Some lipoproteins visible in the images, but lower contamination as compared to GBM Pt3 patient (Figure S9). (C) Western Blot of the plasma EV isolation and the corresponding plasma derivative for GBM Pt4 patient. 50 µL of ‘as is’ plasma lysate was loaded in the plasma lane and 50 µL of concentrated EV lysate was loaded in the EV lane. EVs were obtained from 500 µL plasma isolated through the Izon column and subsequently concentrated using Amicon Ultra 2 mL (10 kDa MWCO). Positive expression of CD9 detected on the EVs and significant reduction of ApoA1 lipoprotein levels in the EV product compared to the corresponding plasma derivative (collected prior to SEC), despite the 10× higher initial volume of plasma used for the EV lane. As expected, no calnexin detected on the EVs and corresponding plasma derivative. (D) Representative image of two GBM CTCs that were identified in the blood of a GBM patient using TSA staining of STEAM (with TSA‐AF488) and of a WBC that was identified by using CD45+CD11c antibody cocktail (with TSA‐AF594). (E) Numbers of CTCs/mL of blood that were identified in the blood of 29 GBM patients (GBM Pts) and 11 healthy donor (HD) controls. Each patient's blood was run through the CTC‐iChip, and the CTC product was plated into a slide. For each patient, the number of CTCs/mL was obtained by counting all the CTCs across the entire slide and by dividing by the initial blood volume. (F) Number of CTCs/mL of blood identified in the GBM patients grouped into two categories: Alive and Deceased. These groups were defined according to the patient's status 12 months post‐tumour resection. (G) Representative image of a substrate with EVs isolated from the plasma of a GBM patient, where the vesicles were stained using TSA for STEAM (with TSA‐AF488) and a cocktail of CD9‐CD81 (with TSA‐AF594). (H) Table showing the total numbers of GBM CTCs and potential GBM EVs identified in the blood and plasma of a select number of GBM patients: five among those presented in Figure 7B,C. The two columns on the left (under ‘CTC‐iChip (whole slide)’) represent the total number of GBM CTCs identified in each patient's blood sample and the number of CTCs normalised by the volume of blood analysed (n. CTCs/mL blood). For this analysis, the cell numbers over an entire glass slide, where all the CTCs obtained from the CTC‐iChip were plated, were considered. The two columns on the right (under ‘Izon SEC (sampling four FOVs)’) depict the number of GBM EVs, defined as the particles double positive for CD9‐CD81 and STEAM, identified from the matched plasma samples of the corresponding GBM patients and the normalised percentages of GBM EVs. The latter was calculated by dividing the number of GBM EVs by the number of total EVs (CD9‐CD81 positive particles) and by subtracting the percentage of STEAM‐positive particles detected for the healthy control. For this analysis, the EV numbers over four FOVs were considered, thus representing a sampling of the total plating area.

Although cell‐derived EVs and cell lines provide excellent model systems for the optimization and validation of fluorescent staining protocols, true utility is seen using clinical samples that are deeply complex. As such, we assessed the feasibility of using our signal amplification strategy for the staining of CTCs and EVs from clinically obtained blood and plasma samples. For this pilot study, blood samples were collected from patients suspected to have glioblastoma at the Massachusetts General Hospital in Boston and from healthy donors. Patients were consented using our IRB protocols (DFCI #05‐300 and MGB #2009‐P‐000295) with institutional oversight. All potential GBM blood samples were collected by trained personnel prior to their first tumour resection, when the tumour burden was predicted to be highest. Following tumour resection, all patients included in this study were ultimately diagnosed with glioblastoma, and subsequently went on to receive treatment with radiation and chemotherapy (Temozolomide, TMZ). For CTC collection, the blood samples were run through the CTC‐iChip (Karabacak et al. 2014), a device co‐developed in our lab for the isolation of CTCs. This microfluidic technology uses a negative selection approach, using fluidic forces and magnetophoresis to deplete the blood samples of red blood cells (RBCs), proteins, circulating nucleic acids and WBC. We selected this approach as it is only one of two known strategies for GBM CTC isolation (Swennenhuis et al. 2016; van Schaijik et al. 2019). With EVs being the primary end goal of this study, we wanted to demonstrate the universal nature of our single EV staining protocol using one of the most common strategies for EV isolation: SEC. This method allows the separation of EVs from other particles based on their size as they pass through a column packed with a porous polysaccharide resin. For a select number of patients, we had matched plasma samples for a paired EV analysis. Plasma samples were obtained by the initial centrifugation (1100 × *g* for 10 min at RT) of paired blood samples collected in vacuum PPT tubes, and were subsequently run through the SEC columns to isolate EVs (500 µL input volume and 1.6 mL purified collection volume). Following isolation, the plasma EV samples were characterised by NTA, WB and TEM. As presented in Figures 7A and S9, NTA confirmed the presence of EV‐sized particles in all patient samples. TEM and WB, which were performed on two representative patient samples (Pt3 and Pt4), revealed the presence of particles with EV morphology and expressing EV markers (CD9), as well as some interpatient variability in the amounts of lipoproteins in the final EV product. Specifically, EV isolation from Pt4 (Figure 7B) had less lipoprotein contamination than the one from Pt3 (Figure S9). However, for both samples WB revealed a significant reduction of ApoA1 levels in the EV isolation compared to their corresponding plasma derivatives (Figures 7C and S9), despite the 10× higher initial volume of plasma used for the EV lane as compared to the plasma lane.

For the staining experiments, both CTCs and EVs were immobilized onto glass substrates following the same protocols utilised for the GBM cell lines and stained using the developed TSA method. For the analysis of CTCs, the developed two‐plex TSA protocol was applied to identify the possible presence of GBM CTCs (via the use of the STEAM cocktail), to discriminate these cells from other cells present in circulation (via the simultaneous use of the WBC cocktail), and to count their numbers across all patients. For the analysis of EVs, the developed TSA protocol was used to identify general EVs (via the use of the CD9‐CD81 cocktail) and to calculate the possible presence and percentage of GBM EVs coming from the brain tumour (via the use of the STEAM cocktail). The choice of first identifying EVs based on the tetraspanins was necessary due to the frequent presence of contaminants in plasma, such as lipoproteins, which share the EV size range but can cause cross‐reactivity with targeted markers. However, it is important to note that the substrate areas available for analysis differed between CTCs and EVs due to distinct imaging procedures. Specifically, for CTCs, the entire cell plating area (325 mm^2^) could be analysed due to the automated scanning of the Vectra microscope system, allowing a comprehensive enumeration of all the CTCs present in each sample. For the EVs, instead, only a limited number of FOVs (four, corresponding to 0.072 mm^2^) could be acquired per sample due to the manual acquisition procedure of the confocal microscope. This approach resulted in the complete evaluation and enumeration of CTCs but only a reduced sampling of all the vesicles. Nonetheless, care was taken to ensure that the sampled areas for EVs were representative of the entire plating areas. We note these differences to highlight that we are not imaging all of the tumour EVs in a sample and accordingly, our results do not reflect a direct comparison between the absolute number of EVs and CTCs within a sample.

As shown in Figure 7D,E, the use of the STEAM cocktail was able to identify the presence of GBM CTCs in 59% of the GBM patients (17 out of 29). Imaging conditions were set such that 0 CTCs/mL were identified in our paired healthy controls (HD, *N* = 11, 0 CTCs/mL, Figure 7E). Moreover, this cocktail specifically stained the CTCs, showing no cross‐reaction with the WBCs present in the same samples, which only showed positivity for CD45 + CD11c (Figure 7D), or with the cells present in the healthy controls. On average, the GBM patients presented ∼6 CTCs/mL (or 45 CTCs/7.5 mL), with their actual counts spreading from ∼1 to 56 CTCs/mL (or 7.5–420 CTCs/7.5 mL). When grouping these patients into two categories, Alive or Deceased, based on their 12‐month post‐diagnosis outcome, the data (Figure 7F) showed a higher range of CTCs/mL (∼2–43) for the Deceased group than for the Alive one (∼1–28). However, the difference between the two groups was not statistically significant (*p* value = 0.09).

For the paired EV analysis, matched plasma samples were available for five of the 29 GBM patients presented in Figure 7E. Figure 7G illustrates a representative image of a substrate with EVs isolated from the plasma of one GBM patient and stained for STEAM and CD9‐CD81. The tables in Figures 7H and S10 report both the CTC and EV counts detected for these five GBM patients. As indicated by the ‘N. total EV’ column in Figure S10, the EV data showed a considerable variation in the number of general EVs (from ∼70 to 1920 over four FOVs), defined as the particles positive for CD9‐CD81, even though the initial plating concentration, in terms of particles/mL as measured by NTA, was the same across the different samples.

Among the EVs that were positive for the analysed tetraspanins (considered as 100% for each sample, Figure S10), a variable number of EVs was also double positive for STEAM (Figure 7H, ‘N. GBM EV’ column). However, only two GBM patients (Pt3 and Pt4) showed a higher percentage of GBM‐EVs (STEAM‐positive EVs) than the paired healthy control, whose number of vesicles positive for STEAM was set as a baseline for positive detection. All the other GBM patients (Pt1, Pt2 and Pt5) presented a lower number of STEAM‐positive EVs than the healthy control and, therefore, their GBM‐EV counts were considered zero. When comparing the number of GBM CTCs/mL blood (Figure 7H, third column from left) with the % GBM EVs double positive for STEAM and CD9‐CD81 (Figure 7H, fifth column from left), the results obtained with cells and EVs followed a similar trend, despite the lower sampling area considered for the vesicles. In particular, patient Pt4 showed the highest numbers of both CTCs/mL blood and potential GBM EVs, followed by patient Pt3, while the other patients showed very low numbers of CTCs/mL blood and the absence of potential GBM EVs compared to the healthy control.

## 4 Discussion

Over the past decade, increasing research has been focused on developing and optimizing techniques for the analysis of single particles in liquid biopsies. These approaches can help better understand the mechanisms driving disease formation and progression (Bordanaba‐Florit et al. 2021). In the context of single CTCs and EV analysis, examining the presence and composition of these particles in biofluids can help identify subpopulations responsible for specific biological functions, such as tumour progression or treatment response (Willms et al. 2018). However, in many cases, single particle analyses are limited by the poor sensitivities of the current analytical methods, which struggle to detect rare ‘events’ in circulation, such as CTCs, or low‐abundance markers, such as many cancer‐specific proteins carried by EVs. Additionally, many sensitive tools for single particle analysis, such as direct stochastic optical reconstruction microscopy (dSTORM), Raman spectroscopy, transmission electron microscopy (TEM) and so forth, require expensive initial investments and extensive user training.

In this work, we try to overcome some of the challenges of single particle analysis by devising an innovative tyramide probe‐based IF amplification technique, which is capable of amplifying low signals. The developed TSA method requires the use of a confocal microscope for imaging, a technology that is far more accessible and cost‐effective than a TEM or a dSTORM imager. Although dSTORM offers high sensitivity and superior molecular localization at the nanoscale, the method requires the use of fluorescent antibodies with a specific blinking behaviour in combination with an appropriate buffer solution that efficiently regulates such blinking. In contrast, our TSA approach utilises unconjugated primary antibodies that can be flexibly paired with various TSA fluorescent probes and does not require specialised buffer conditions, making it a simpler and more versatile alternative to dSTORM. Compared to flow cytometry, which enables high‐throughput, quantitative analysis and population‐level measurements, TSA offers distinct advantages in detecting low‐abundance markers due to its signal amplification capability. Flow cytometry, in contrast, lacks this amplification, making it less sensitive to weak signals. Additionally, TSA requires lower antibody concentrations, provides stable signals suitable for multiple imaging sessions, and allows signal strength adjustments by varying the incubation time of tyramide probes, making it particularly advantageous for single‐particle analysis. Furthermore, compared to the other single‐EV approaches based on conventional IF (Guo et al. 2021; Hilton and White 2021; Jeong et al. 2023; Jeong, Son, Tae, et al. 2023; Lee et al. 2018; Zhou et al. 2020), our TSA method offers the unique advantage of enhanced fluorescent signals through the sole use of antibodies and without the need for complex nanostructured substrates. Moreover, the presented approach demonstrates multiplexing, a capability that has not been shown in the other single EV studies employing tyramide probes for signal amplification (Chen et al. 2022; Nguyen et al. 2022; Reynolds et al. 2023), and does not require vesicle lysis, keeping the EVs in their native form. These features make the protocol affordable and facilitate the detection, analysis and co‐localization of rare and low abundant markers that would otherwise be barely or not detected. Despite these advantages, achieving optimal IF for EVs on a confocal microscope, including multiplexing capabilities, requires meticulous effort and has seldom been rigorously explored. Further, it is even more rare to have EVs and cells investigated in both model systems and clinically relevant patient samples.

A common challenge in single particle analyses is the potential presence of clumps and aggregates, which can introduce artifacts and compromise the accuracy of the method. To minimise this risk, we optimised the plating concentrations of both cells and EVs based on the total binding surface to favour the immobilization of predominantly single particles. In addition, we implemented several mitigation strategies. For CTC immobilization, the two‐step PFA fixation prior to and after cytospin reduced aggregation, provided the best preservation of cell morphology, and improved the staining specificity. This step, combined with optimised plating concentration, resulted in predominantly single‐cell immobilization, as shown in Figure 2. However, for the fever cells that still agglomerated, we employed the analysis algorithm to manage them. Specifically, when two cells were merely touching, the algorithm could segment and analyse them individually based on shape. In contrast, larger clumps or merged objects were excluded from analysis to avoid introducing bias. For EV immobilization, we decreased the possibility of aggregates by using freshly isolated samples as increased aggregates were observed in frozen preparations. We also analysed the distribution of the fluorescent signals detected for each EV at both tested concentrations. The stable intensity distribution across the two conditions suggests that we successfully immobilised and analysed mostly single EVs. If EV aggregates had been present at the highest EV concentration, we would have observed a noticeable shift of the distribution toward higher fluorescence intensities. As for CTCs, we also tuned the analysis parameters to exclude abnormally large or irregularly shaped fluorescent signals, likely representing aggregates, or to separate them if they appear to stem from closely spaced individual EVs.

The validation results obtained with GBM cell lines (Figure 2) show the possibility of applying the developed TSA protocol for the specific staining of single CTCs. Our focus on CTCs from glioblastoma stems from our lab's primary interests and the significant potential associated with analysing these cells. Given the challenges in accessing the brain, examining cells that can cross the blood‐brain barrier (BBB) and enter the circulation represents a minimally invasive and invaluable method to get information about this aggressive cancer. However, research on this topic is limited due to the rare presence of CTCs in peripheral blood, likely due to the poor ability of GBM cells to intravasate in the absence of BBB disruption or the apparent paradox of rare systemic metastases in this highly invasive and angiogenic cancer (Sullivan et al. 2014). To overcome this challenge, the TSA protocol presented in this study is supported by our technology for CTC isolation (CTC‐iChip) (Karabacak et al. 2014), which enables the enrichment of these rare circulating cells. The specificity of staining achieved by using a cocktail of antibodies further strengthens the effectiveness of the developed TSA protocol, as NSB typically increases with the number of antibodies used. However, our findings indicate that our protocol, when paired with a careful selection of antibodies, is highly effective in minimizing NSB.

When compared with the conventional approaches of DS and PSS (Figure 2), the developed TSA protocol presents amplified fluorescent signals and much broader signal dynamic ranges. These features allow for a better marker comparison across multiple samples and enable the detection of cells with a lower marker expression, which would otherwise be at or below background levels when stained using conventional methods like DS. Confirming this aspect is the fact that, when staining GBM cells for EGFR using DS (Figure 2A, left), the fluorescent signals are so weak that they are comparable to the background levels and the negligible WBC autofluorescence. Additionally, the data obtained with GBM cells suggest successful multiplexing of up to two tyramide probe colours (plus DAPI) without the need for a quenching buffer between the two staining cycles. This result is likely due to the fact that, for cells, the endogenous HRP from the first secondary antibody (used to target STEAM) is mostly used or deactivated when the second staining cycle is performed. Moreover, such multiplexed staining was specific whether it was used to separate different cell subpopulations (e.g., CTCs from WBCs) or label multiple markers on the same cells (e.g., STEAM vs. CD9‐CD81).

When applied to EVs, the results confirm specific staining achieved with the developed TSA protocol, along with the expected amplification of the fluorescent signals and dynamic ranges, and the possibility for multiplexing. As visible in Figures 4 and S5, the signal amplification obtained with PSS and TSA significantly improved the detection limits. Higher EV counts/FOV (>4000) could be detected as compared to the DS approach (<100) despite using the same EV plating concentration. Interestingly, while the PSS method showed intermediate signal intensities, it presented higher EV counts/FOV (∼7500) than the TSA approach (∼4500). This behaviour was likely due to some EVs in the TSA method being ‘obscured’ by the halo of nearby stronger EV signals. However, we believe that this effect can be minimised by optimizing the plating concentration of the vesicles onto the substrate to increase the minimum distance between adjacent particles. On the other hand, these findings also suggest that if the marker of interest does not need significant amplification, PSS represents a valuable alternative approach to TSA. Multiplexing represents a significant advancement in the TSA staining of single EVs, since this aspect has not been investigated in the other EV studies employing tyramide probes for signal amplification. Our protocol successfully detected two colour probes on individual vesicles, enabling the identification of both generic EV markers, such as the tetraspanins, and tumour‐specific markers, such as those targeted by the STEAM cocktail.

When considering the LOD, our method demonstrates the capabilities to detect single low‐abundance markers on single EVs, as both EGFR and Met could be separately detected. Moreover, the data showed significant differences in the expression levels between the two EV samples (GBM1 and GBM2). While EGFR expression on the EVs was consistent with the CTC data and its amplification on GBM1 EVs could be confirmed by two complementary methods (SNaPshot and immunoaffinity capture), EV Met expression was opposite to what was observed in CTCs. This result may reflect some biological differences in protein packaging between cells and their secreted vesicles, which may vary depending on the specific cell line. In terms of the minimum numbers of proteins per EVs detectable by our method, we would like to emphasise that the detection thresholds obtained in this study are not universal. They will greatly depend on multiple factors, including the specific antibody‐protein pair, antibody affinity, epitope accessibility and probe efficiency. While our approach offers a practical estimation of the detection limit, it does not support absolute quantification. A more rigorous and comprehensive analysis would be required for that purpose, which is beyond the scope of the present study.

A significant difference compared to the staining of cells regards the stability of the EV fluorescent signals over time, with the TSA and PSS methods significantly outperforming DS (Figure 5C, I). This characteristic is related to the much lower abundance of proteins on the surface of single EVs compared to cells, which results in much dimmer fluorescent signals. Moreover, the data highlight the importance of introducing a quenching buffer between the two TSA staining cycles to minimise cross‐reactivity between colours during multiplexed measurements on EVs. Due to the nature of the TSA staining, it is crucial to choose primary and secondary antibodies that do not cause cross‐reactions when two or more colours are used. This issue can easily arise if (i) the secondary antibody of the second staining cycle cross‐reacts with the primary antibody of the first cycle, or (ii) the TSA probe of the second staining cycle reacts with the HRP of the secondary antibody of the first cycle. A proper choice of antibodies can prevent the first issue. However, when staining EVs with the developed TSA protocol, the results suggested that the second issue could be faced (Figure 6G). In the absence of a quenching buffer, the HRP was not fully deactivated after the first staining cycle, probably due to the lower degree of EV staining compared with cells. Therefore, a quenching buffer was essential to properly deactivate such HRP and ensure specificity for the second staining colour.

Although positive results were obtained with cell lines and cell‐derived EVs, the true utility of a method lies in its successful application to real patient samples and its ability to address clinical questions. To explore the clinical relevance of the developed TSA method, we applied it to paired analyses of CTCs and EVs collected from glioblastoma patients at a clinically significant time point: before tumour resection, when patients were treatment‐naive, and the tumours were presumed to be at their maximum burden. The choice of these samples provides insights into the behaviour of this cancer and helps us assess whether and how many CTCs and tumour EVs could be detected in the peripheral blood prior to the major disruption of the BBB by surgery, thus exploring the potential of these biomarkers for liquid biopsies. These two features make this study innovative as, to the best of our knowledge, it is the first preliminary investigation to simultaneously assess paired CTCs and EVs samples from GBM patients at this specific time point.

The results obtained for this pilot study were promising. The data on cells showed that the developed protocol identified CTCs in the blood of most GBM patients analysed, suggesting that GBM cells can enter the systemic circulation and survive there long enough to be detected. The average number of CTCs/mL of blood (∼6 CTCs/mL, or 45 CTCs/7.5 mL), was equivalent to that of many other solid tumours (Saylor et al. 2024), which is a bit surprising given the presence of the BBB. However, it has been demonstrated that for this aggressive tumour, inflammation and an overall diseased state promote a ‘leakiness’/partial disruption of the BBB, permitting these rare cells to pass through (Clarke and Chang 2012; Liu et al. 2010). Notably, by using the presented TSA method, the identification of GBM CTCs was significantly improved compared to our previous study (Sullivan et al. 2014), where higher false positives (0–7 CTCs/mL or 0–53 CTCs/7.5 mL) were detected in the healthy donor controls. With our new TSA protocol, we were able to reduce the number of CTCs/mL for all our paired healthy controls to zero, setting this number as the new imaging threshold for CTC detection. This approach increased our GBM CTC detection rate to 59%, almost double the one obtained in our previous study (30%) (Sullivan et al. 2014). It should be noted that in our prior study, patients were included at all stages of treatment, further highlighting the gains in detection using our new staining approach.

When working with EVs derived from these patients, we employed several strategies to preserve the quality of the samples and reduce lipoprotein contaminations. Specifically, all plasma samples were aliquoted and underwent only one freeze‐thaw cycle. All blood, and therefore plasma, samples were collected prior to surgery, after fasting periods of 8–14 h, which likely reduced the presence of larger lipoproteins in some of the patients. Despite TEM images showed some degrees of lipoprotein contaminations in the final EV product, WB revealed a significant reduction of ApoA1 levels in the EV fractions compared to their corresponding plasma derivatives. These finding suggested that our SEC and centrifugation protocol was effective in reducing lipoprotein content, acknowledging that inter‐patient variability persists and that a combination with other methods such as density gradient ultrafiltration might further improve the final EV purity. We also implemented measures within our TSA method to minimise false positive signals. First, our antibody‐based approach targeted markers not typically found on lipoproteins, reducing the chance of nonspecific staining. Moreover, only particles that were double positive for both the tetraspanins and STEAM cocktail were considered as EVs. This strategy should further limit the likelihood of signals derived from lipoprotein or other contaminants that could get co‐isolated when isolating EVs from plasma samples using SEC. Overall, the results were promising but suggested the need for further optimization. The TSA method identified particles positive for both STEAM and CD9‐CD81 markers. The number of tetraspanin‐positive particles per FOV was relatively low (<250 for most patients), despite a higher concentration being expected based on the plating concentration estimated from NTA. This result likely suggested the presence of non‐EV particles in the samples or the absence/low abundance of the specific tetraspanins analysed (CD9‐CD81). Moreover, our paired healthy control also presented a fraction of EVs positive for the STEAM cocktail. Since this healthy donor had no brain tumour, their value of STEAM‐positive EVs was set as a baseline for positive GBM EV detection and brought the number of STEAM‐positive EVs to zero for most GBM patients. Because of this adjustment, only patients Pt3 and Pt4 showed potential GBM EVs above threshold values. Although not optimal, these results were not totally surprising considering that GBM EVs likely represent a very small fraction of the total EVs in plasma and that very low numbers of CTCs/mL blood were detected for all these patients except for Pt4. However, the staining data showed a similar percentage of GBM EVs for the two samples that showed positive signals (Pt3 and Pt4), which were the same analysed by WB and TEM. Since these samples had different lipoprotein contamination levels, the data suggest that our TSA method specifically targeted mostly vesicles and not lipoproteins or other contaminants.

Despite all the promising results, the current study has some limitations that warrant further investigation. First, we optimised our TSA approach for the multiplexed staining of up to two colours, a lower capability compared to other currently available IF approaches. Although higher levels of multiplexing are theoretically possible, they may be constrained by steric hindrance from multiple amplified TSA probes, which could saturate or block the limited surface area of single EVs. Second, despite the optimization of the EV immobilization strategy, the stability of the fluorescent signal distribution across different concentrations, and the image processing, a small possibility of aggregates being recorded persists, especially for the smallest particles below the diffraction limit of light. Third, while our approach enables a practical estimation of the number of proteins detectable per EV, it does not support absolute quantification. Additionally, although the protocol can be readily adapted to stain cells and EVs from other types of cell lines, diseases, or patients, this study was limited to a focused set of markers and a small number of samples from GBM patients. When comparing the detection efficiency of CTC and EVs in terms of number of patients with GBM‐positive particles above threshold levels, our results seem to suggest a lower efficiency of the method for EVs. We believe this finding may be due to the substantially smaller area analysed for the EVs (four FOVs) compared to the whole slide used for CTC analysis. However, this aspect should be further investigated in future studies, and efforts should be made to enhance EV detection efficiency if it continues to be lower than that of CTCs. Finally, we believe that the isolation and analysis of GBM EVs can be significantly improved by both combining SEC isolation with other methods such as density gradient ultrafiltration and by integrating the developed protocol with our Herringbone chip (HB‐chip), a microfluidic device developed in our lab for the isolation of EVs from cancer patients with high specificity and sensitivity (Reátegui et al. 2018). By using an optimised cocktail of antibodies specifically tailored for GBM, the HB‐chip is expected to improve the capture efficiency of GBM EVs, enabling a more robust and precise analysis of these particles compared to their analysis directly from bulk plasma.

## 5 Conclusions

In conclusion, we present the development of a TSA‐based immunofluorescent protocol for the staining of single CTCs and EVs. The method relies solely on the use of unlabelled primary and HRP‐secondary antibodies, followed by labelled tyramide probes, for detection. The application of this protocol to CTCs and EVs isolated from GBM cell lines demonstrates significant amplification of fluorescent signals, broader dynamic ranges and greater signal stability compared to conventional methods using labelled primary or secondary antibodies. Moreover, it is the first time that a tyramide probe‐based IF amplification method shows multiplexing on single EVs targeting both general and tumour‐specific markers. To explore the clinical relevance of the developed method, we applied our TSA protocol to paired CTC and EV samples isolated from patients with GBM. Although the immediate clinical utility remains to be established, the data suggest specific staining of GBM CTCs via the use of the STEAM cocktail and the presence of these cells in circulation, giving an insight into their intravasation prior to the disruption of the BBB by surgery. Despite the need for further optimization, the results with EVs indicate the presence of vesicles positive for tetraspanins and GBM‐specific markers, generally correlating with the counts of CTCs for most analysed patients. In addition to further optimizing the identification of GBM EVs from clinical plasma samples, in the future, we plan to increase the multiplexing capabilities of the presented TSA protocol, integrate the staining protocol with our HB‐chip‐based capture of the EVs, and expand the cohort of patients and diseases that can be analysed by our method. Furthermore, it would be interesting to explore the optimization and adaptation of the TSA protocol for flow cytometry, in order to potentially combine the strengths of both techniques to enhance sensitivity and scalability within a single analytical platform.

## Author Contributions

The specific contributions of each author are outlined below using the Contributor Roles Taxonomy (CRediT): Sara Cavallaro: conceptualization, data curation, formal analysis, funding acquisition, investigation, methodology, validation, visualization, writing – original draft, writing – review and editing. Sara Veiga: investigation, validation, visualization, writing – original draft, writing – review and editing. Raheel Ahmad: investigation, validation, visualization, writing – original draft, writing – review and editing. Berent Aldikacti: conceptualization, formal analysis, methodology, investigation, validation. Mollie Bienstock: formal analysis, investigation. Diane Capen: investigation, visualization. Daniel C. Rabe: resources, validation. Uyen Ho: data curation, project administration, writing – review and editing. Dasol Lee: investigation, resources. Daniel A. Ruiz‐Torres: methodology, validation. Hiroaki Wakimoto: resources, investigation, validation, writing – review and editing. Jorg Dietrich: funding acquisition, resources, project administration. Brian V. Nahed: funding acquisition, resources, project administration. Shannon L. Stott: conceptualization, funding acquisition, methodology, project administration, resources, supervision, writing – original draft, writing – review and editing.

## Conflicts of Interest

The authors declare the following conflicts of interest: Shannon L. Stott serves as a scientific advisory board member for Streck, LLC, unrelated to this work. All other authors declare they have no competing interests.

## Supporting information




**Supporting Information**. The following files are available free of charge.
Supporting information containing further details on the developed staining protocol and supplementary figures not included in the main manuscript text.

MIBlood‐EV form containing all relevant information about blood and plasma collection and processing.

## Data Availability

Data available on request from the authors.
