# Supplementary material for: Signal Amplification for Fluorescent Staining of Single Particles in Liquid Biopsies: Circulating Tumour Cells and Extracellular Vesicles
Source: J Extracell Vesicles. 2025 Oct 8;14(10):e70167. doi: 10.1002/jev2.70167 (PMC12508258; doi:10.1002/jev2.70167)
Supplement: Supplementary file 1 — Supporting Information. The following files are available free of charge. Supporting information containing further details on the developed staining protocol and supplementary figures not included in the main manuscript text. [file JEV2-14-e70167-s002.docx]

**Supplementary Information for**

Signal amplification for fluorescent staining of single particles in liquid biopsies: circulating tumor cells and extracellular vesicles

*Sara Cavallaro, Sara I. Veiga, Raheel Ahmad, Berent Aldikacti, Mollie Bienstock, Diane Capen, Daniel C. Rabe, Uyen Ho, Dasol Lee, Daniel A. Ruiz-Torres, Hiroaki Wakimoto, Jorg Dietrich, Brian V. Nahed, and Shannon L. Stott*

**Materials and Methods**

*Complete protocol for the TSA staining of EVs.*

Note: All the steps are performed on a rocker, keeping the slide inside a dark box.

1. TNT washing x3 (2 quick + 1x 1 min);
2. Pre-block with IgG antibodies (Goat Anti-Mouse IgG1 Fab Fragment and Goat Anti-Rat IgG Fab Fragment, 45 min) to reduce NSB of the primary antibodies;
3. TNT washing x2 (2 quick);
4. Block with Antibody Diluent/Block x2 (2x 5 min);
5. TNT washing x3 (1 quick + 2x 5 min);
6. Incubation with first primary antibody cocktail diluted in TNB solution (STEAM cocktail, 1 h);
7. TNT washing x3 (2 quick + 1x 5 min);
8. Incubation with first HRP-secondary antibody cocktail diluted in TNB solution (Anti-Mouse IgG2b HRP + Anti-Mouse IgM HRP, 45 min);
9. TNT washing x3 (2 quick + 1x 5 min);
10. Incubation with first TSA probe (TSA-AF488) diluted in 1x Plus Automation Amplification Diluent (5 min);
11. TNT washing x1 (1 quick);
12. Quench with 3% H_2_O_2_ solution (20 min);
13. TNT washing x1 (5 min);
14. Block with Antibody Diluent/Block x2 (2x 5 min);
15. TNT washing x3 (1 quick + 2x 5 min);
16. Incubation with second primary antibody cocktail diluted in TNB solution (CD9-CD81, 1 h);
17. TNT washing x3 (2 quick + 1x 5 min);
18. Incubation with second HRP-secondary antibody cocktail diluted in TNB solution (Anti-Mouse IgG1 HRP, 45 min);
19. TNT washing x3 (2 quick + 1x 5 min);
20. Incubation with second TSA probe (TSA-AF594) diluted in 1x Plus Automation Amplification Diluent (5 min);
21. TNT washing x3 (2 quick + 1x 5 min);
22. PBS washing x3 (2 quick + 1x 5 min);
23. Withdrawal of most PBS leaving a thin PBS layer on the well, removal of the Millicell chamber, mounting with coverslip and sealing with nail polish.

*Protocol for the TSA staining of CTCs:*

1. Follow “Complete protocol for the TSA staining of EVs” until step 21, skipping steps 12-13 due to the absence of cross-reaction between the first and second staining cycles.
2. Block with Antibody Diluent/Block x2 (2x 5 min);
3. TNT washing x3 (1 quick + 2x 5 min);
4. Incubation with DAPI solution (20 min);
5. TNT washing x3 (2 quick + 1x 5 min);
6. DI water washing (1 quick);
7. Drying of the slide in air, mounting with a coverslip using PBS or mounting media and sealing of the coverslip with nail polish.

*Adjustments of the TSA protocol for direct (DS) and primary + secondary (PSS) staining:*

- For DS: skip steps 8-12 and 18-21 of the TSA staining protocol of EVs, and replace steps 6 and 16 with the incubation of the fluorescent primary antibody cocktails, labeled with AF488 and AF594, respectively.
- For PSS: skip steps 10-12 and 20-21 of the TSA staining protocol of EVs, and replace steps 8 and 18 with the incubation of the corresponding fluorescent secondary antibody cocktails, labeled with AF488 and AF594, respectively.

*Composition of TNT washing solution and TNB diluent solution.*

1. **TNT washing solution**: 0.1M Tris-HCl, pH 7.5; 0.15M NaCl; 0.3% (for CTCs) or 0.01% (for EVs) Triton X-100.
2. 1M Tris-HCl: 50mL
3. 5M NaCl: 15mL
4. Milli-Q Water: bring total volume to 500mL
5. 100% Triton X-100: 1.5mL for CTCs or 50 µL for EVs
6. Stir solution on plate to mix well for 20 min and filter it. Store at 4°C for up to 1 month.
7. **TNB diluent solution**: 0.1M Tris-HCl, pH 7.5; 0.15M NaCl; 0.5% Blocking Reagent; 0.3% (for CTCs) or 0.01% (for EVs) Triton X-100
8. 1M Tris-HCl: 50mL
9. 5M NaCl: 15mL
10. Milli-Q Water: to 500mL
11. Place beaker on hot plate; heat gradually to 55°C while slowly stirring in 2.5g blocking reagent. Stir for 25 minutes until blocking reagent dissolved.
12. Store in 50mL aliquots at -20°C.
13. After thawing aliquot, add 150 µL for CTCs (or 5 µL for EVs) Triton X-100 to the 50mL volume. Vortex, rock for 10min, and filter it using a bigger cap filter.
14. Store thawed aliquots for up to 1 month at 4°C.

*Concentrations of the staining antibodies used in this study.*

- **Antibodies for initial blocking**: same for CTC and EV staining
  - Goat Anti-Mouse IgG1 Fab Fragment: 50 µg/mL
  - Goat Anti-Rat IgG Fab Fragment: 50 µg/mL
- **Primary antibodies**:
  - STEAM cocktail for CTCs and EVs:
    - Sox2: 1 µg/mL
    - Tubulin: 1 µg/mL
    - EGFR: 1 µg/mL
    - A2B5: 0.25 µg/mL
    - MET: 1 µg/mL
  - Cocktail for WBC staining only:
    - CD45: 1 µg/mL
    - CD11c: 1 µg/mL
  - Tetraspanins for EVs staining only:
    - CD9: 1 µg/mL
    - CD81: 1 µg/mL
    - CD63: 2 µg/mL
- **Secondary antibodies**: same for CTC and EV staining. This is the final concentration of the specific antibody in the cocktail.
  - HRP-conjugated antibodies for TSA:
    - Anti-Mouse IgG2b HRP: 1 µg/mL
    - Anti-Mouse IgM HRP antibody: 1 µg/mL
    - Anti-Rat IgG2b HRP: 1 µg/mL
    - Anti-Mouse IgG1 HRP antibody: 1 µg/mL
  - Fluorescently labeled antibodies for PSS:
    - Goat Anti-Mouse IgG2b-AF488: 1 µg/mL
    - Goat Anti-Mouse IgG1-AF594: 1 µg/mL
- **TSA probes for TSA**: same for CTC and EV staining
  - TSA-AF488: 100x dilution from stock concentration
  - TSA-AF594: 100x dilution from stock concentration
- **DAPI:** for CTC staining only
  - DAPI: 1000x dilution in TNB from stock (A) solution, where stock (A) solution is given by adding 2.1 ul of DAPI stock solution (5 mg/mL) to 100 µL of PBS

*Matching of the primary and secondary antibodies for the various staining strategies*

- **CTC staining**:

| **Primary antibody** | **Primary antibody species** | **Secondary antibody** | **Secondary antibody species** | **TSA probe** |
| --- | --- | --- | --- | --- |
| Sox2 | Mouse IgG2b | Anti-Mouse IgG2b HRP + Anti-Mouse IgM HRP | Goat + Goat | TSA-AF488 |
| Tubulin | Mouse IgG2b |  |  |  |
| EGFR | Mouse IgG2b |  |  |  |
| A2B5 | Mouse IgM |  |  |  |
| Met | Mouse IgG2b |  |  |  |
| CD45 | Rat IgG2b | Anti-Rat IgG2b HRP + Anti-Mouse IgG1 HRP | Goat + Goat | TSA-AF594 |
| CD11c | Mouse IgG1 |  |  |  |

- **EV staining**:

| **Primary antibody** | **Primary antibody species** | **Secondary antibody** | **Secondary antibody species** | **TSA probe** |
| --- | --- | --- | --- | --- |
| Sox2 | Mouse IgG2b | Anti-Mouse IgG2b HRP + Anti-Mouse IgM HRP | Goat + Goat | TSA-AF488 |
| Tubulin | Mouse IgG2b |  |  |  |
| EGFR | Mouse IgG2b |  |  |  |
| A2B5 | Mouse IgM |  |  |  |
| Met | Mouse IgG2b |  |  |  |
| CD9 | Mouse IgG1 | Anti-Mouse IgG1 HRP | Goat | TSA-AF594 |
| CD81 | Mouse IgG1 |  |  |  |

**Table S1.**

| Gene | Log2 copy number ratio (log2cnr) | Copy number conversion (2^log2cnr^) |
| --- | --- | --- |
| MDM2 | 1.49 | 2.80 |
| EGFR | 1.30 | 2.46 |
| CDK4 | 2.13 | 4.38 |
| MYC | 1.21 | 2.31 |

**Table S1**. SNaPshot data obtained on the MGG72 cell line, here referred to as GBM1, showing amplification of EGFR. Specifically, the log2cnr represents the relative copy number of the gene compared to a normal reference genome and its positive value suggests an increase in the copy number of the gene, thus an amplification. A log2cnr of 1.3 indicates that the copy number of EGFR in this cell line is 2.46 times the normal number of copies.

**Figure S1.**

**
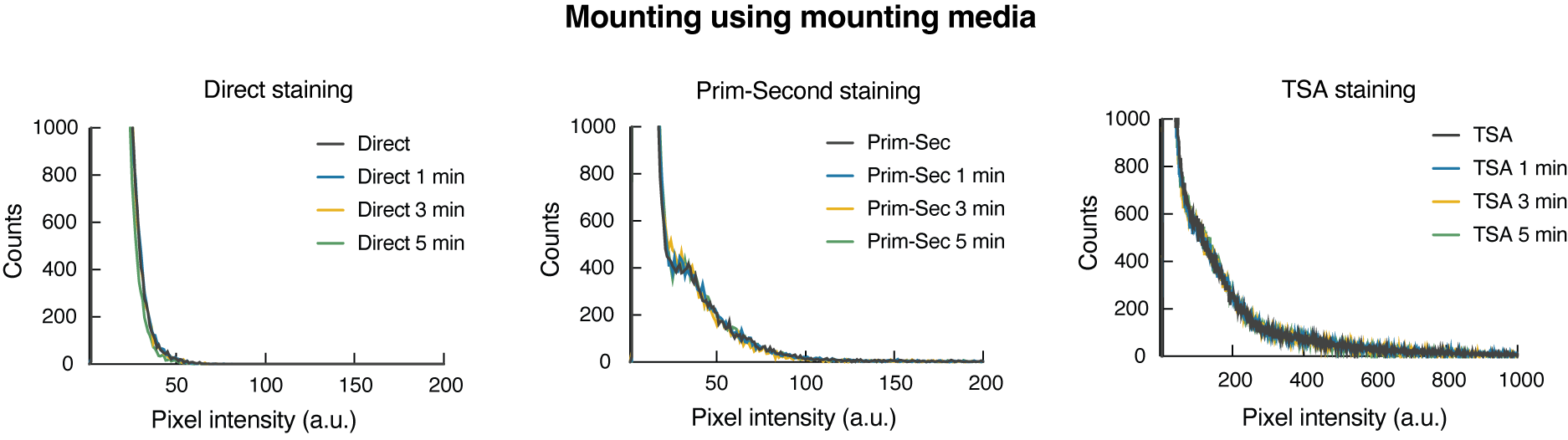
**

**Figure S1**. Time dependence of EGFR signal over a 5-minute period for the three staining strategies. The intensity of each pixel (x-axis, measured in arbitrary units, a.u.) positive for EGFR was considered for this analysis. The y-axis represents the number of EGFR-positive pixels having a specific intensity. The coverslips were mounted on top of the glass slides with the CTCs using the Prolong Antifade Mounting media, which creates a stabilization as well as an amplification of the fluorescence signals. Slides were imaged using a confocal microscope. The immobilized CTCs that were stained for EGFR were constantly excited with a laser for 5 minutes, and snapshot images were captured at time 0 and after 1, 3, and 5 minutes, respectively.

**Figure S2.**


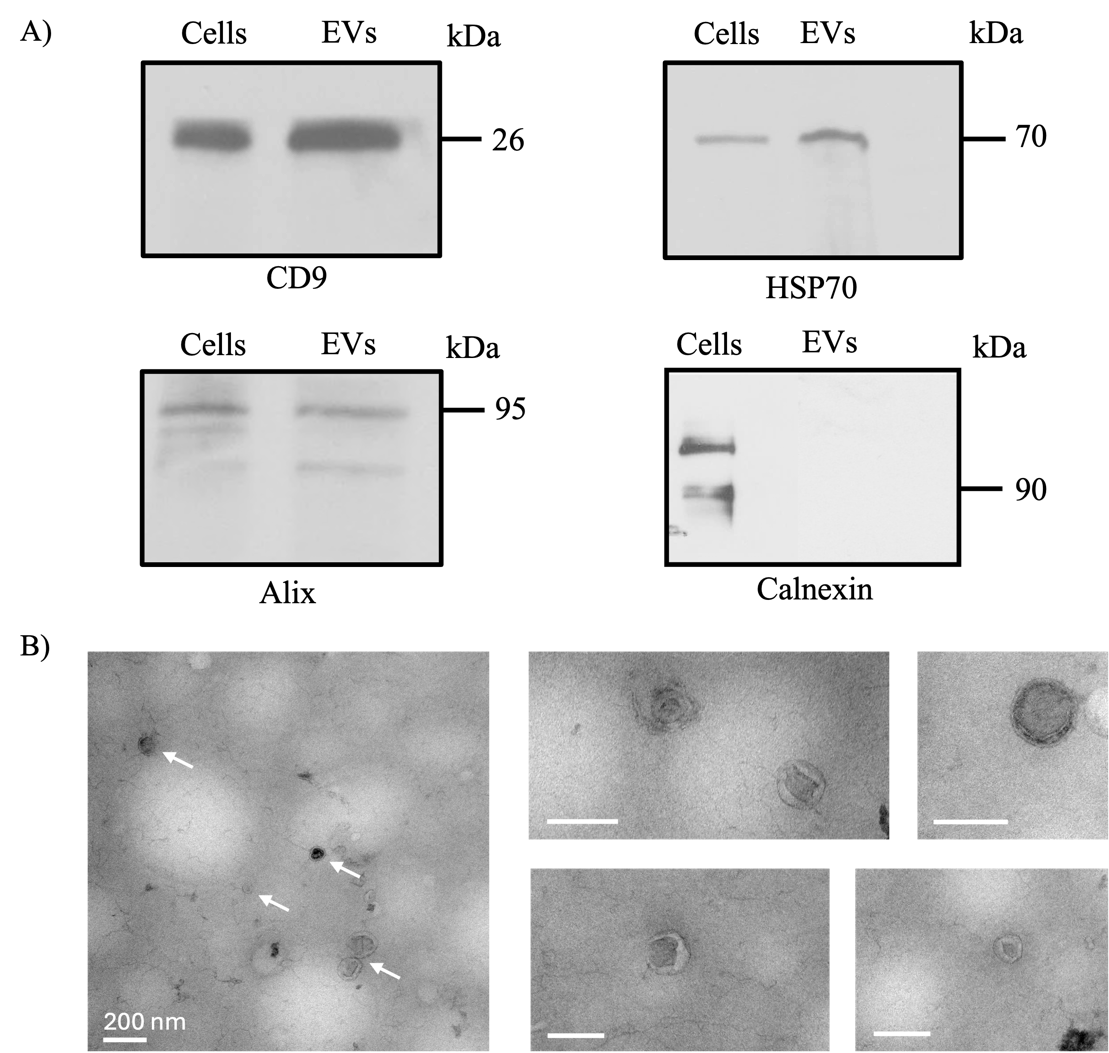


**Figure S2**. Additional characterization of the EVs isolated from GBM cell lines. (A) Immunoblotting performed on the GBM2 parental CTCs (Cells) and isolated EVs to test the presence of CD9, HSP70, Alix and Calnexin. 4 µg of proteins from the cell lysate and the EV lysate were loaded for the cells and EVs lanes, respectively. As visible, both CTCs and EVs show positive expression of CD9, Alix and HSP70, with an enrichment of CD9 and HSP70 on EVs as compared to CTCs. Furthermore, the EV isolate shows the absence of cellular contamination via Calnexin, which only shows a positive detection on the CTCs. (B) Representative transmission electron microscopy (TEM) images showing vesicles enclosed by a lipid bilayer in the size range of EVs. EVs having various sizes were detected. The images also suggested the absence of major contaminants in the isolated EV samples.

**Figure S3**.


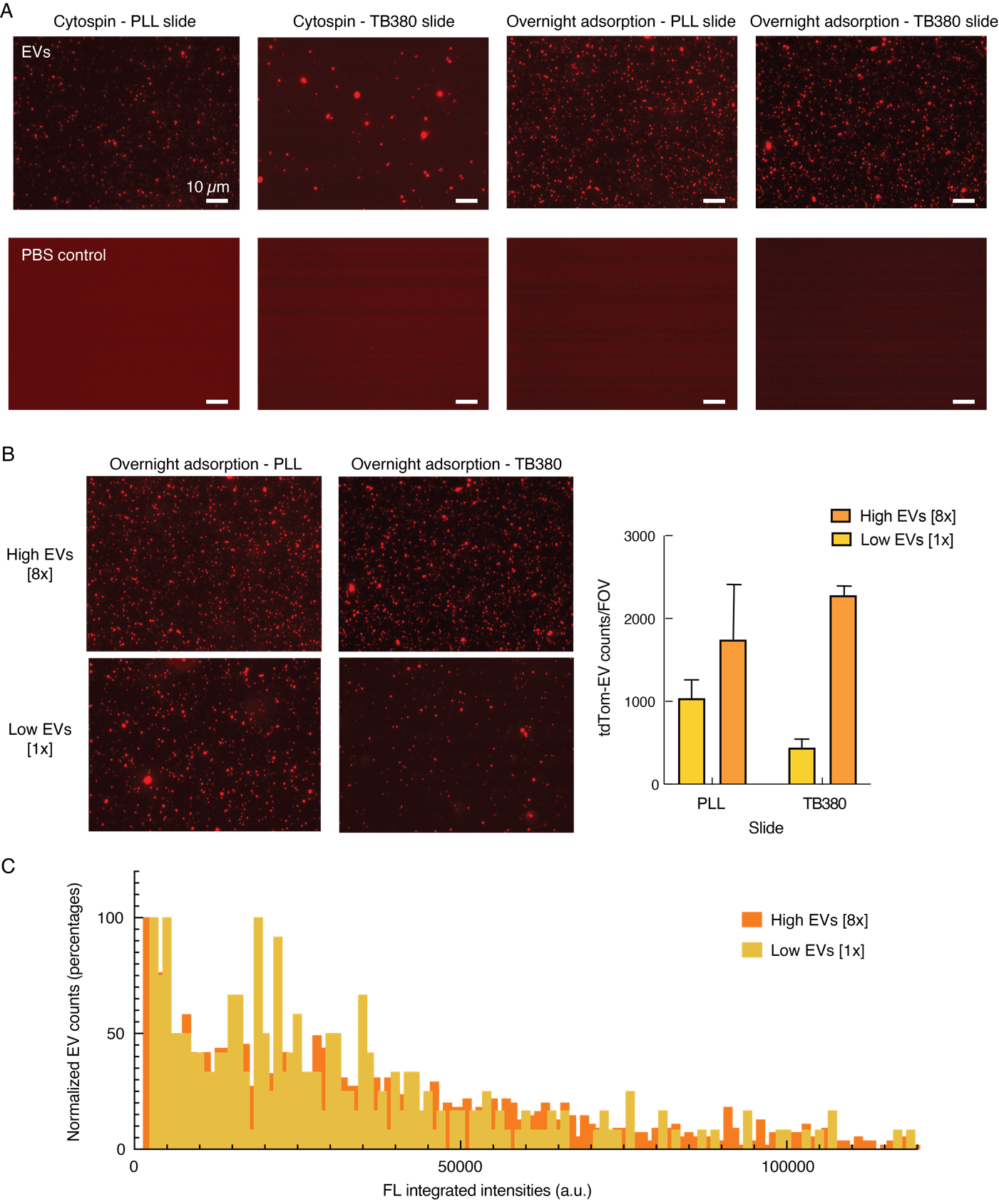


**Figure S3**. (A) Representative images of the tdTom-EVs that were captured onto two different glass slides, PLL and TB380, using either cytospin or overnight adsorption. Representative images of the respective PBS control substrates included for comparison. Scale bar represents 10 µm in all images. (B) Representative images and corresponding tdTom-EV counts/FOV of the EVs that were captured onto the two glass types (PLL and TB380) using overnight adsorption only, for the two different tdTom-EV concentrations analyzed [1x] and [8x]. These counts were obtained considering 4 FOVs for each slide type and vesicle concentration. (C) Normalized distribution of the FL signals of all the single spots detected as EVs, calculated as the integrated intensities over all the pixels forming each single EV, for the two concentrations tested (Low EVs [1x] and High EVs [8x]). The EV counts on the y-axis were normalized by considering the peak value of each distribution as 100% and the zero value as 0%. As visible, the distribution of the FL signals for the high EV concentration remained similar to that of the low EVs (p-value NS), likely indicating that we are predominantly immobilizing and analyzing single EVs.

**Figure S4**.


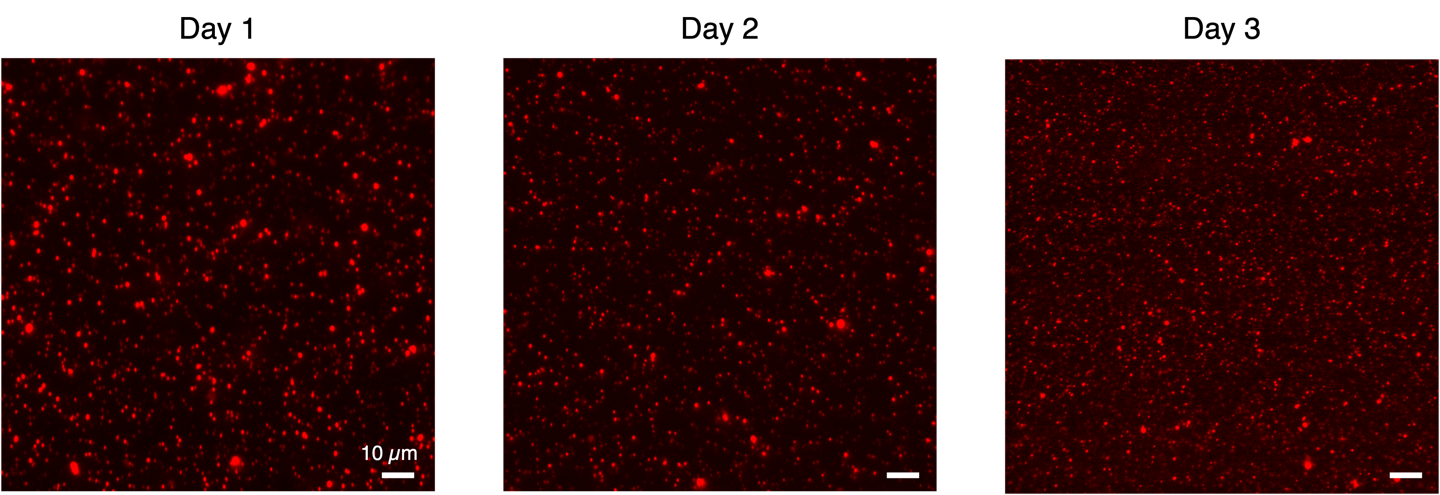


**Figure S4**. Representative images of the tdTom-EVs that were captured onto the TB380 slides using overnight adsorption over a period of 3 days. As shown, the EVs remain stably immobilized onto the substrates for at least 3 days. Scale bar represents 10 µm.

**Figure S5**.


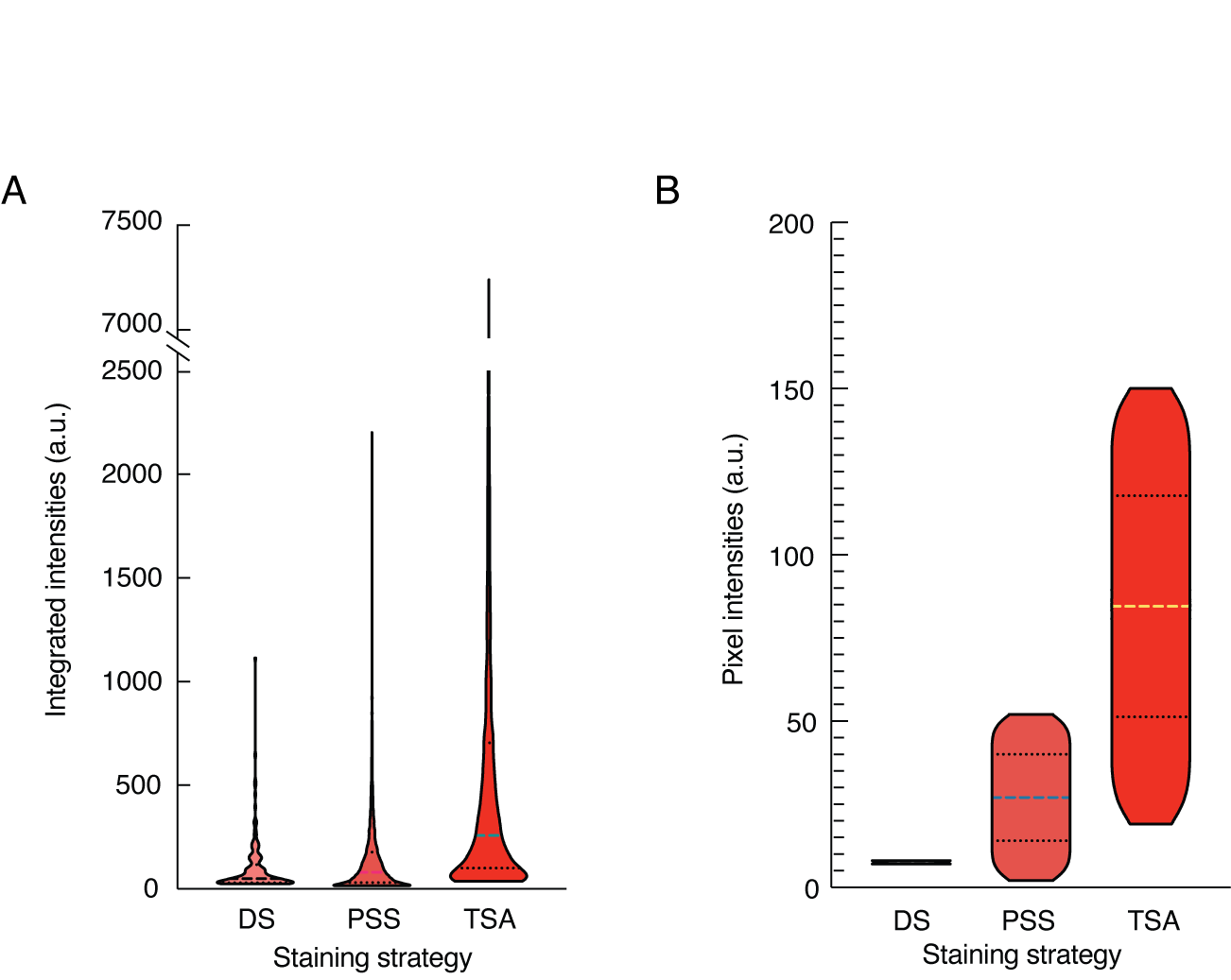


| **Dynamic Range Intensity Type** | **DS** | **PSS** | **TSA** |
| --- | --- | --- | --- |
| Integrated intensity (IntI) | 1080 | 2200 | 7150 |
| Pixel intensity (PixI) | 4 | 48 | 130 |

**Figure S5**. Distributions of the (A) Integrated intensities and (B) Pixel intensities for the three techniques analyzed, Direct Staining (DS), Primary Secondary Staining (PSS) and Tyramide Signal Amplification (TSA). Table shows the dynamic ranges for the two types of intensities (IntI and PixI) and the three techniques analyzed (DS, PSS, TSA).

**Figure S6**.


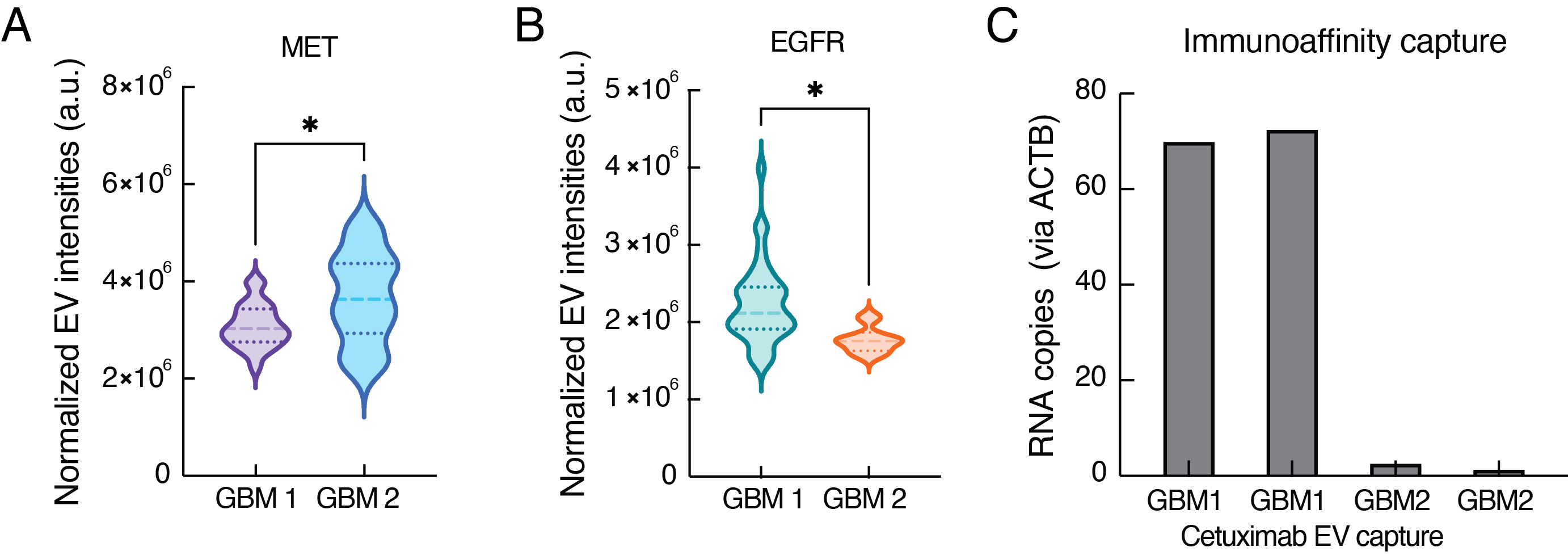


**Figure S6.** (A-B) TSA staining of MET and EGFR proteins on single EVs isolated from GBM1 and GBM2 cell lines. Both proteins were targeted using a TSA-AF488 probe. The normalized EV intensities shown on the y-axis represent the normalized mean FL intensity distribution of single EVs. (C) Immunoaffinity capture of the same GBM EVs into a microfluidic device using Cetuximab as a capture probe (which targets EGFR protein on the EVs surface). Number of EVs captured inside the device measured via the RNA analysis of their ACTB content. As visible, more GBM1 EVs were capture inside the device compared to GBM2 EVs. Being the EVs captured via EGFR, it is possible to infer that these vesicles express more EGFR than GBM2 vesicles, confirming the staining data. For each EV sample, the two bars represent duplicate experiments.

**Figure S7**.


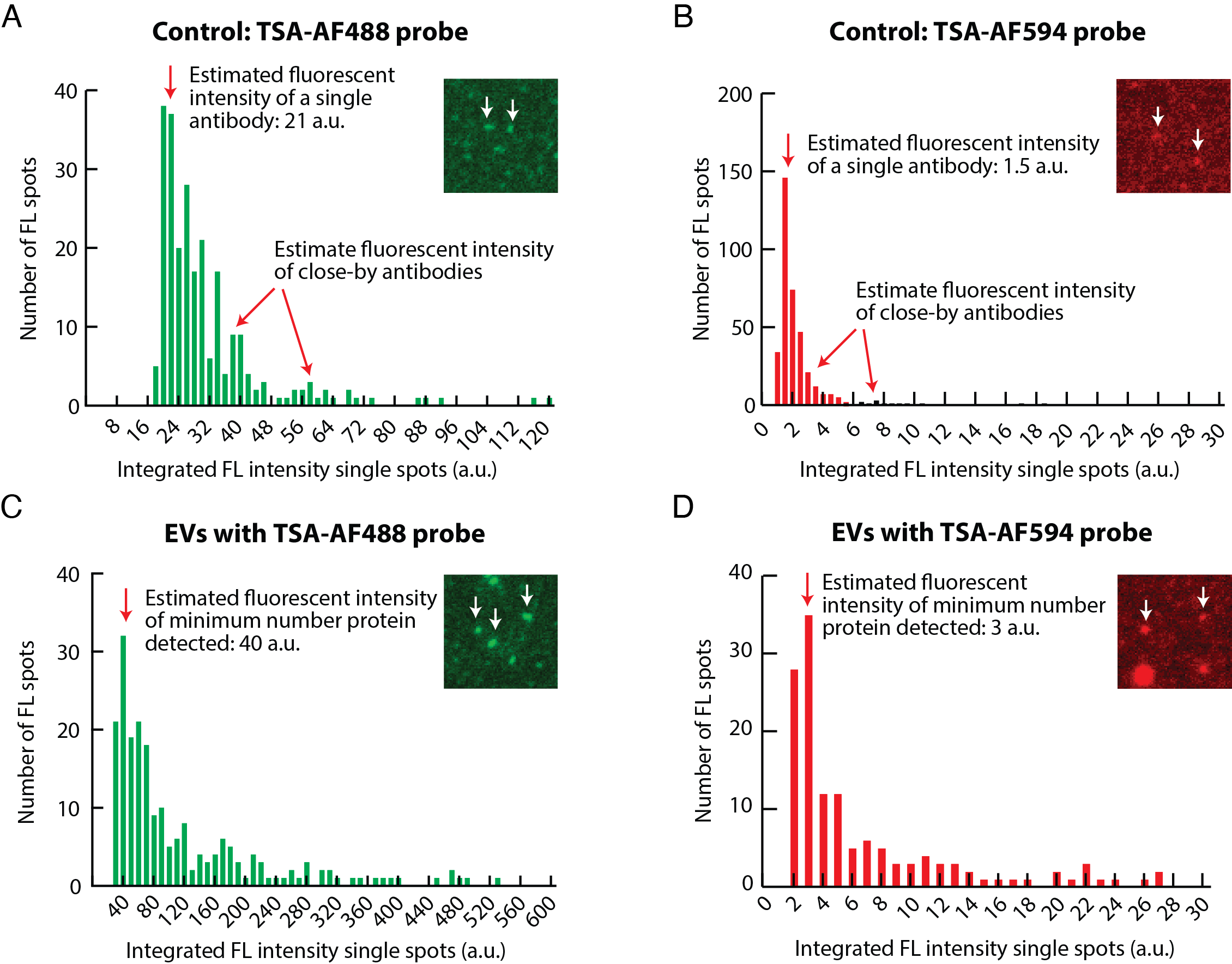


| **TSA probe** | **Estimated intensity of single antibody-probe conjugate (a.u)** | **Intensity of minimum signal detected (a.u)** | **Estimated minimum number of proteins/EV detected** |
| --- | --- | --- | --- |
| **TSA-AF488** | 21 | 40 | 2 |
| **TSA-AF594** | 1.5 | 3 | 2 |

**Figure S7.** Estimation of the limits of detection (LOD) of our TSA staining method in terms of the minimum number of proteins per EV that can be detected as positive. For all the plots, the x-axis represents the integrated fluorescent intensity calculated considering all the pixels forming a single FL spot, while the y-axis represents the number of FL spots detected for each specific intensity. (A) Control substrate, without EVs, with immobilized HRP-conjugated secondary antibodies followed by TSA-AF488 probes, incubated for 5 min. The first peak of the fluorescent intensity distribution was considered as the value of the fluorescent intensity of a single antibody (21 a.u.), when TSA-amplified. Inset shows a representative image of distinct fluorescent spots attributed to single antibodies, pointed out by arrows. (B) Sample substrate with EVs, stained for the STEAM antibody cocktail using the TSA method (TSA-AF488 probe). The first peak of the fluorescence intensity distribution (40 a.u.) was considered the minimum signal detected by our technique and was used to estimate the minimum number of proteins detected per EVs by diving it by the fluorescent intensity of a single antibody obtained in the respective control substrate, Fig. S7A. (C) Control substrate, without EVs, with immobilized HRP-secondary antibodies followed by TSA-AF594 probes, incubated for 5 min. The first peak of the fluorescence intensity distribution was considered as the value of the fluorescent intensity of a single antibody (1.5 a.u.), when TSA-amplified. Inset shows a representative image of distinct fluorescent spots attributed to single antibodies, pointed out by arrows. (D) Sample substrate with EVs, stained for the CD9-CD81 antibody cocktail using the TSA method (TSA-AF594 probe). The first peak of the fluorescence intensity distribution (3 a.u.) was considered the minimum signal detected by our technique and was used to estimate the minimum number of proteins detected per EV by dividing it by the fluorescent intensity of a single antibody obtained in the respective control substrate, Fig. S7B.

**Figure S8**.


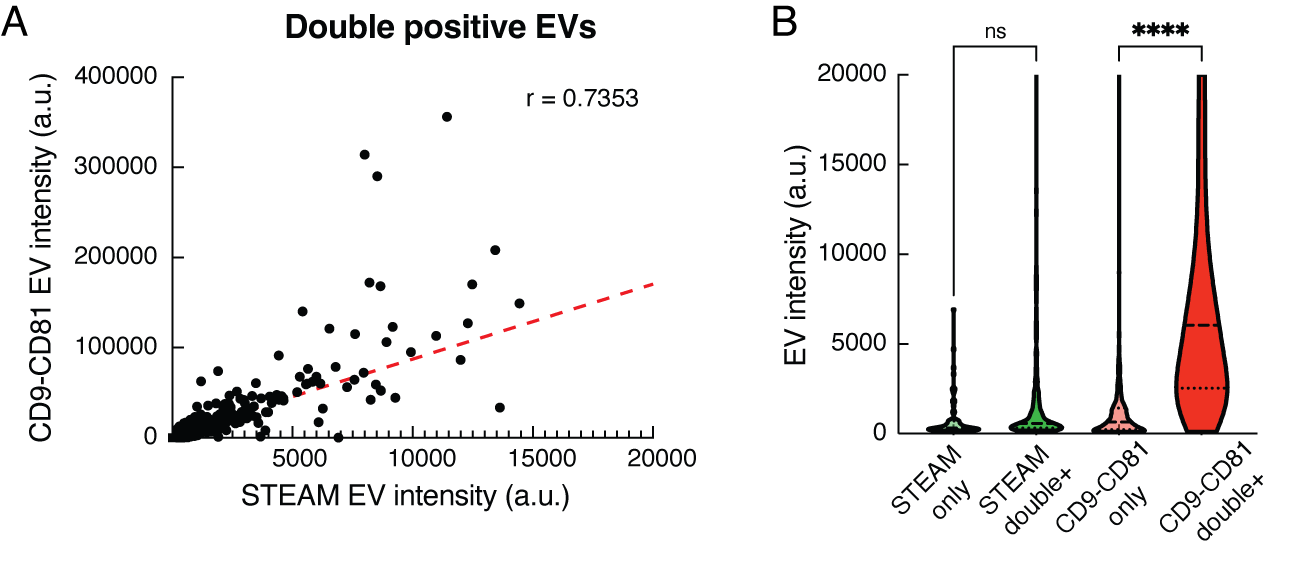


**Figure S8.** (A) Correlation between the expression of our staining cocktail for tumor markers, STEAM, and the expression for general EV markers, CD9-CD81, on single vesicles. Integrated intensities calculated over all the pixels constituting an EVs were plotted in this analysis. ‘r’ represents the correlation coefficient. (B) Distribution of the STEAM and CD9-CD81 integrated intensities of single EVs for the different EV subpopulations, including EVs only positive for STEAM (STEAM only), EVs only positive for CD9-CD81 (CD9-CD81 only) and EVs double positive for both marker combinations (double +). In this latter case, two distributions, one for STEAM (STEAM double +) and one for CD9-CD81 (CD9-CD81 double +), are plotted for the double positive EVs.

**Figure S9.**

**
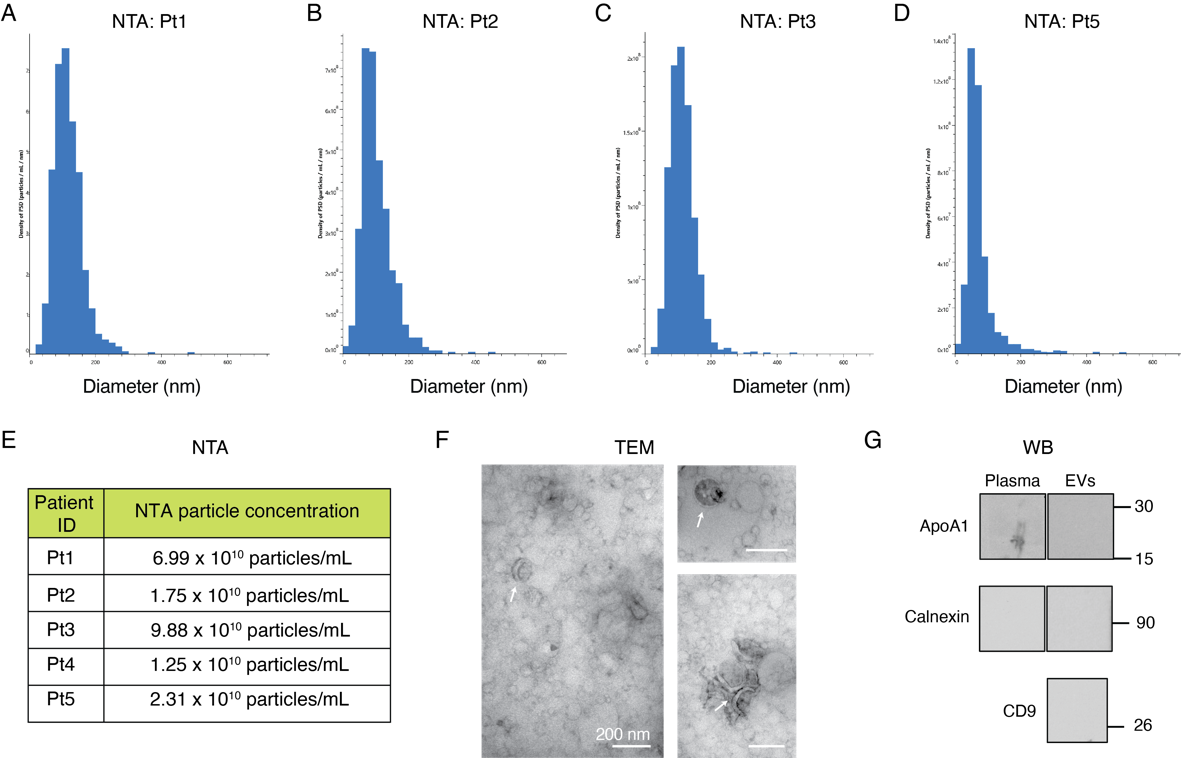
**

**Figure S9.** Additional characterization data of the EVs isolated from the GBM patient plasma samples. (A-D) Particle size distribution obtained for all the EV samples isolated from the GBM patient plasmas by Izon SEC using NTA (ViewSizer 3000, Horiba). (E) Measured NTA total particle concentrations for all the GBM patients analyzed. (F) Representative TEM images of the plasma EVs isolated by SEC from GBM Pt3 patient, showing vesicles enclosed by a lipid bilayer in the size range of EVs (white arrows). This sample showed more lipoprotein contamination than the other plasma sample analyzed by TEM, Pt4 (Figure 7B). (G) Western Blot of the plasma EVs and the corresponding plasma derivative for GBM Pt3 patient. 50 µL of “as is” plasma lysate was loaded in the plasma lane and 50 µL of concentrated EV lysate was loaded in the EV lane. EVs were obtained from 500 µL plasma isolated through the Izon column and subsequently concentrated using Amicon Ultra 2mL (10 kDa MWCO). As expected, no calnexin was detected on the EVs and corresponding plasma derivative, and a significant reduction of ApoA1 lipoprotein levels was detected in the EV product compared to the corresponding plasma derivative (collected prior to SEC). Unfortunately, no CD9 could be detected for this sample, likely due to the lower number of vesicles that showed positivity for CD9 by the staining protocol for this patient as compared to GBM Pt4 patient (Figure s10).

**Figure S10.**

| Patient ID | N. total EVs (CD9-CD81+ EVs) | Norm. % tot. EVs |
| --- | --- | --- |
| Pt1 | 68 | 100 |
| Pt2 | 76 | 100 |
| Pt3 | 216 | 100 |
| Pt4 | 1919 | 100 |
| Pt5 | 219 | 100 |

**Figure S10**. Table showing the total number of general EVs, defined as particles positive for CD9-CD81 tetraspanins, detected in the five GBM patients for which matched plasma samples were available. Data was obtained by counting and summing the particles over four FOVs. For each patient, the total EV number was taken as a reference for the total number of particles (100%) and was used to calculate the percentage of GBM-positive EVs in Figure 7E.
